# Field trials of the novel varroacide, 1-allyloxy-4-propoxybenzene, against *Varroa destructor* in Western Canada

**DOI:** 10.1038/s41598-025-23935-7

**Published:** 2025-11-17

**Authors:** Robert X. Lu, Abdullah Ibrahim, Olav Rueppell, Erika Plettner, Stephen F. Pernal

**Affiliations:** 1 Department of Biology, University of Oxford, Oxford, UK; 2 Beaverlodge Research Farm, Agriculture and Agri-Food Canada, Beaverlodge, AB Canada; 3 Department of Biological Sciences, University of Alberta, Edmonton, AB Canada; 4 Department of Chemistry, Simon Fraser University, Surrey, BC Canada

**Keywords:** Varroa destructor, Honey bees, Varroacide, Efficacy, Pollinator health, Apiculture, Entomology, Parasitology, Chemical biology

## Abstract

*Varroa destructor* is an ectoparasite of western honey bees (*Apis mellifera*), substantially damaging managed colonies worldwide. Various chemical “varroacides” have been developed to help beekeepers combat infestations of these mites. Nevertheless, treatments may induce non-target toxicity in bees and humans, and their application can be laborious and hazardous. Moreover, widespread varroacide usage has seen the emergence of resistant mite populations, necessitating the need for new treatment options. The dialkoxybenzene, 1-allyloxy-4-propoxybenzene (codenamed **3c**{*3*,*6*}), has previously been shown to induce paralysis and death of varroa in laboratory experiments, and capable of reducing varroa infestations in full-sized bee colonies. Our study sought to comprehensively investigate **3c**{*3*,*6*} as a varroacide by optimizing dosages, treatment schedules and application methods across two sets of parallel experiments in Western Canada. Colonies treated with **3c**{*3*,*6*} were compared to untreated negative controls and positive control colonies treated with the thymol-based commercial product, Thymovar. Across all experiments, **3c**{*3*,*6*} had mite-killing efficacy comparable to Thymovar, being consistently greater than the untreated colonies, though efficacy varied between years. Treatment with **3c**{*3*,*6*} did not impact food stores or bee populations. As treatment efficacy varied with dosage and application method, additional studies will be required to optimize **3c**{*3*,*6*} treatment regimens for potential commercial use.

**Supplementary Information:**

The online version contains supplementary material available at 10.1038/s41598-025-23935-7.

## Introduction

The western honey bee, *Apis mellifera* L. (Hymenoptera: Apidae) is the most widely used managed pollinator in the world, pollinating numerous plant species in both wild and agricultural systems^1–3^. These pollination services are vital for ensuring global food security, contributing to up to one-third of all food consumed^4,5^. However, compounding impacts of multiple honey bee stressors – particularly disease-causing pathogens and parasites – have resulted in increasing rates of colony mortality, straining the sustainability of the global beekeeping industry^6–12^.

The most severe parasite of honey bees is the ectoparasitic mite *Varroa destructor* Anderson and Trueman (Mesostigmata: Laelapidae), or “varroa”^13–15^. Direct feeding activity of varroa on honey bee tissues causes weakness, compromised immune systems, and reduced foraging capacity^16–19^. These pathologies (collectively termed “varroosis”) are exacerbated by the suite of RNA viruses vectored by the mite^20,21^. The life cycle of varroa is divisible into two stages: reproductive and dispersal^22–24^. During the reproductive stage, varroa mite foundresses and their developing offspring primarily feed on haemolymph of developing pupae within sealed brood cells^19^. The dispersal stage consists of mature adult foundresses, which situate themselves below the abdominal sclerites of adult bees (typically nurse-age workers within the broodnest) and feed on fat body tissues^17,25–27^. The short generation span (~ 3 weeks) enables rapid expansion of varroa populations within a colony which, in tandem with the induced pathologies, may result in colony loss, especially during winter^28–30^.

Numerous chemical treatments have been developed to control varroa (termed “varroacides”)^31^. These include a diverse array of both synthetic compounds, such as pyrethroids (tau-fluvalinate and flumethrin), organophosphates (coumaphos), and formamidines (amitraz), as well as varroacides derived from natural compounds, including organic acids (especially formic and oxalic acids) and phytochemicals (hop beta acids and essential oils, notably thymol)^32^. However, different varroacides may possess various drawbacks in terms of efficacy, toxicity and treatment application methods^33^. Notably, many varroacidal compounds may leave chemical residues in wax and honey, potentially leading to non-target toxicity in both honey bees and humans^34,35^. While naturally-derived varroacides tend to exhibit relatively low non-target toxicity and have so far remained effective against varroa, their vapour-based mode of action is directly influenced by ambient temperature and humidity which can affect treatment efficacy^36^. Many naturally-derived varroacides also tend to exhibit lower efficacies per individual treatment, requiring repeated applications to eliminate mites^37^. Organic acids especially pose a risk to users, as their corrosive and volatile nature necessitates careful application and the use of personal protective equipment (PPE), including respirators, eye protection, and gloves^23,38^. Moreover, formic acid is also known to increase queen mortality at high dosages^39–41^.

Extensive use of synthetic varroacides has invariably led to the emergence of varroacide-resistant mite populations, resulting in diminished efficacy^42–46^. Long-term sustainable varroa control must therefore employ strategies to reduce the incidence of varroacide resistance, such as through rotation or combination of different treatments^47^. Chemical treatments may be also coupled with non-chemical controls (such as selective breeding or cultural techniques) as part of integrated pest management (IPM) regimens, further disrupting varroa behaviours such as host-finding and mating^48,49^. The development of novel varroacides therefore plays a key role towards sustainable varroa management and the sustainability of beekeeping in general, by expanding the palette of treatment options and the longevity of their effectiveness.

One candidate chemical with demonstrated varroacidal potential is 1-allyloxy-4-propoxybenzene (CAS No. 1005006-02-9), codenamed **3c**{*3*,*6*}^50^. A low-molecular weight dialkoxybenzene, **3c**{*3*,*6*} is a structural mimic of phytochemical odorants, such as 1,4-dimethoxybenzene, and was first explored as a feeding deterrent against cabbage looper caterpillars (*Trichoplusia ni* (Hübner))^51,52^. In vitro topical assays of **3c**{*3*,*6*} applied to varroa mites rapidly induced paralysis and death, while sublethal levels reduce mites’ host-finding abilities and cause arrestment during host-finding^50,53^. Compared to structurally-similar dialkoxybenzenes, **3c**{*3*,*6*} possesses greater and more rapid varroacidal activity^50^. The toxic effects of **3c**{*3*,*6*} do not appear to occur in exposed honey bees, whose behaviours remain unchanged^50,53^. Initial hypotheses posited **3c**{*3*,*6*} to act as a cholinesterase inhibitor, though experimental evaluations found no clear evidence of this mode of action^50^. Thus, while **3c**{*3*,*6*} does not appear to be a cholinesterase inhibitor, its specific mode of action remains unclear. The potential of compound **3c**{*3*,*6*} to persist in the environment is low as it is biodegradable, being readily metabolized by common strains of the cosmopolitan soil bacterium *Pseudomonas putida* Trevisan^54^.

Two parallel in situ field trials of **3c**{*3*,*6*} were carried out in 2019 in Beaverlodge, Alberta (AB) and in Surrey, within the Fraser Valley of British Columbia (BC), Canada. These evaluated the efficacy of **3c**{*3*,*6*} for reducing varroa infestations within a commercial apiary setting^50^. A dosage of 5 g **3c**{*3*,*6*} per colony was applied during the fall via ten compound-impregnated tongue depressors suspended between brood frames of varroa-infested colonies over 28 days, followed by a six-week clean-up treatment with the amitraz-based varroacide, Apivar (3.33% amitraz active ingredient, a.i.). Results showed that **3c**{*3*,*6*} effectively lowered varroa populations, with efficacies of 81.1 ± 2.9% and 51.2 ± 6.2% (mean ± SE) in AB and BC, respectively. In addition, **3c**{*3*,*6*}-treated colonies in AB had lower overwintering mortality (40%) than the untreated negative control colonies (100%).

The current study built upon the previous field trials to further evaluate the efficacy of **3c**{*3*,*6*} as an in situ varroacide. We carried out two sets of parallel experiments in Beaverlodge, AB and Surrey, BC, which spanned through the late summer and early fall of 2021 and 2022. These experiments also aimed to quantify the mortality-based efficacy of **3c**{*3*,*6*} in comparison with Thymovar (15 g thymol/wafer), which functioned as a positive control. Across the two experimental seasons, study designs were modified to optimize the amount of the compound applied to honey bee colonies and its delivery method.

## Results

### Impact of **3c***{3*,*6}* treatment on varroa infestation levels

**Fig. 1:**
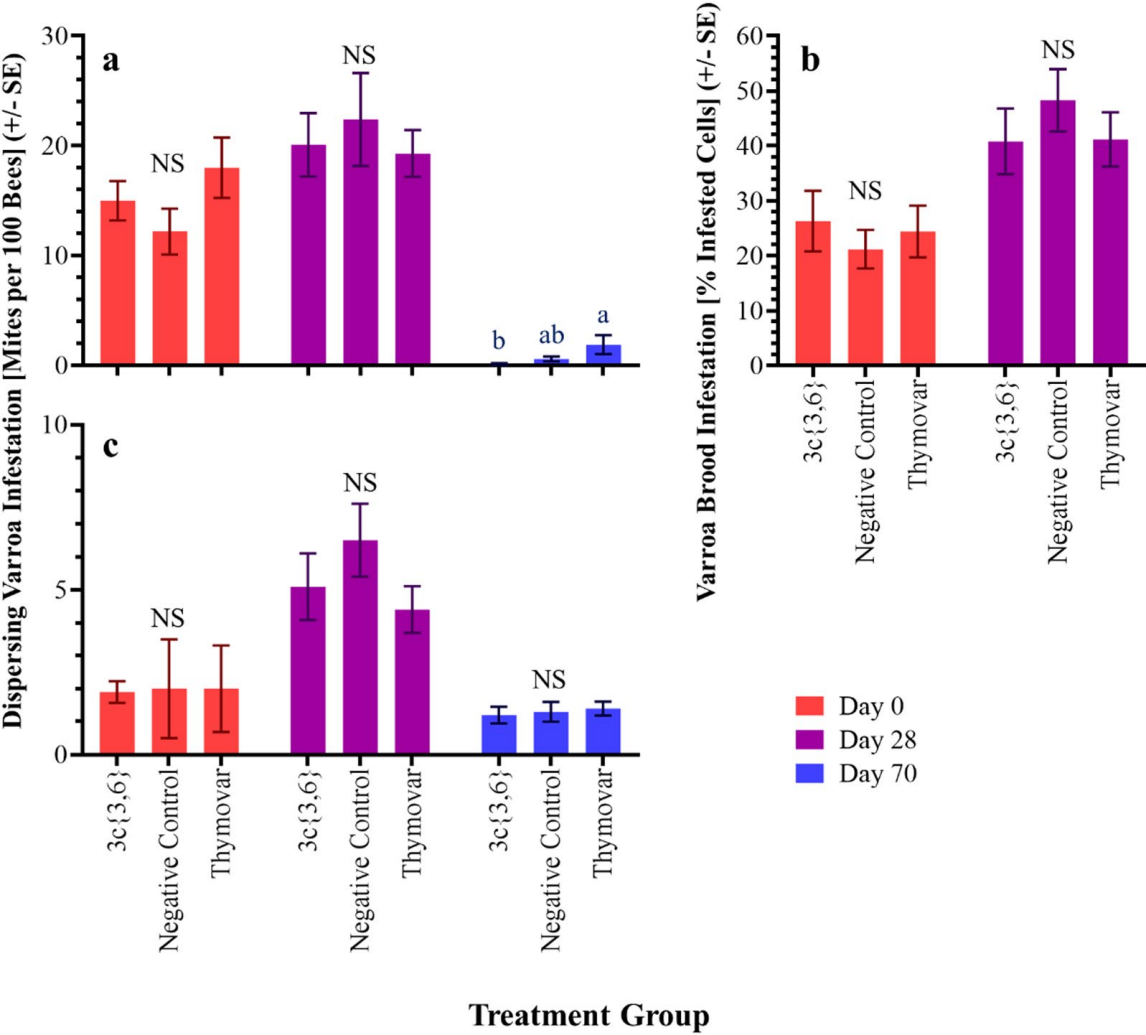
Dispersing phase and capped brood varroa mite (*Varroa destructor*) infestation levels in the 2021 experiments. (**a**) Dispersing phase mite infestations in AB 2021 colonies. (**b**) Capped brood infestations in AB 2021 colonies. (**c**) Dispersing phase mite infestations in BC 2021 colonies. Values represent mean ± SE. Different letters denote significant post-hoc pairwise differences (α = 0.05; NS, not significant; see Supplementary Table S1). For BC colonies in 2021, varroa infestations in capped brood were not measured on Day 0, and too few colonies had sufficient brood on Day 28 to permit meaningful comparisons. As such, these results are not presented. AB = Alberta; BC = British Columbia.

In the 2021 experiments, over all colonies, initial (Day 0) mite infestation levels on adult bees were 15.0 ± 1.3% (mean ± SE) in AB (Fig. 1a), and 2.0 ± 0.2% in BC (Fig. 1c). Initial infestations were statistically similar among treatment groups in AB (*F* = 1.67; df = 2, 27; *P* = 0.206) and BC (*H* = 0.11; df = 2; *P* = 0.946) (Supplementary Table S1). No statistical differences in dispersing mite levels on Day 28 were observed between treatments in AB (*H* = 0.94; df = 2; *P* = 0.626) and BC (*F* = 1.24; df = 2, 27; *p* = 0.307). On Day 70, **3c**{*3*,*6*}-treated colonies in AB showed significantly lower levels of dispersing mites than Thymovar-treated colonies, while infestation levels in untreated negative control colonies were higher than the **3c**{*3*,*6*} group and lower than the Thymovar group, but not statistically different from either (*H* = 6.33, df = 2, *P* = 0.042); BC colonies remained statistically similar (*H* = 2.76; df = 2; *P* = 0.252).

In 2021, Day 0 varroa infestation levels in capped brood were 24.0 ± 2.6% in AB (Fig. 1b), being similar among treatment groups (*F* = 0.31; df = 2, 27; *P* = 0.738), and were not monitored in BC. On Day 28, capped brood infestation levels in AB continued to remain similar among treatments, having increased to 43.4 ± 3.1% overall (*F* = 0.58; df = 2, 27; *P* = 0.566), while too few colonies in BC had sufficient brood to permit meaningful between-group comparisons.

**Fig. 2:**
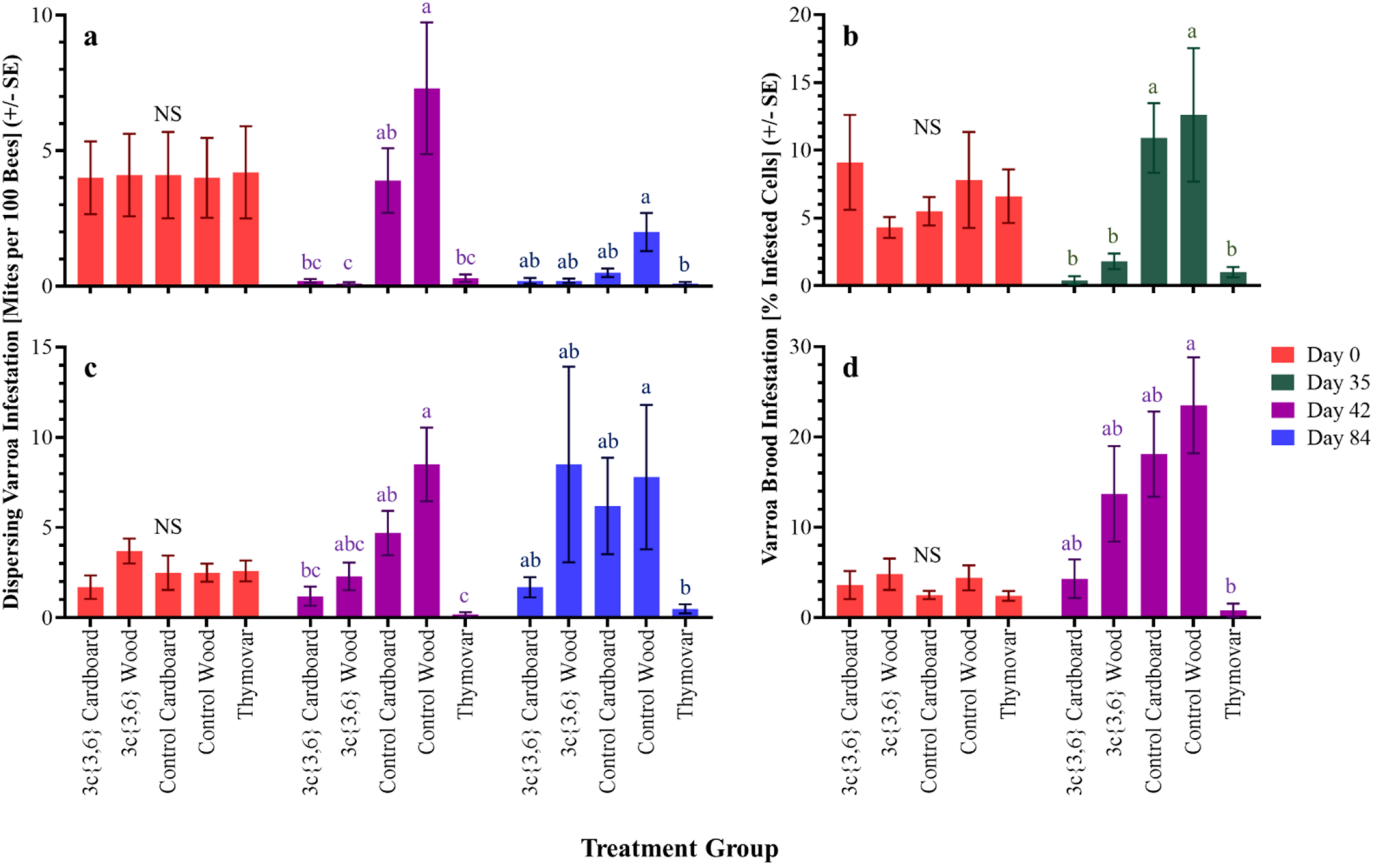
Dispersing phase and capped brood varroa mite (*Varroa destructor*) infestation levels in the 2022 experiments. (**a**) Dispersing phase mite infestations in AB 2022 colonies. (**b**) Capped brood infestations in AB 2022 colonies. (**c**) Dispersing phase mite infestations in BC 2022 colonies. (**d**) Capped brood infestations in BC 2022 colonies. Values represent mean ± SE. Different letters denote significant post-hoc pairwise differences (α = 0.05; NS, not significant; see Supplementary Table S2). AB = Alberta; BC = British Columbia.

In the 2022 experiments, Day 0 mite infestations on adult bees were 4.1 ± 0.7% in AB (Fig. 2a), and 2.6 ± 0.3% in BC (Fig. 2c), with levels being similar among treatment groups for both AB (*H* = 0.03, df = 4, *P* > 0.999) and BC (*H* = 7.00, df = 4, *P* = 0.136) (Supplementary Table S2). On Day 42, dispersing mite levels in AB differed significantly among treatment groups (*H* = 23.85; df = 4; *P* < 0.001), with **3c**{*3*,*6*}-wood, **3c**{*3*,*6*}-cardboard and Thymovar-treated colonies having significantly lower mean mite levels than both wooden and cardboard untreated negative control groups. Nevertheless, mite levels in the untreated negative cardboard control group were not statistically different from the **3c**{*3*,*6*}-cardboard and Thymovar-treated colonies (Fig. 2a). In BC, colonies also had significant differences in Day 42 dispersing mite infestations among groups (*H* = 21.13; df = 4; *P* < 0.001) (Fig. 2c), with Thymovar-treated colonies having the lowest infestation levels, being significantly smaller than both untreated negative controls with high infestations, and the two **3c**{*3*,*6*} treatments being statistically similar to Thymovar-treated and untreated negative control colonies. It was noted that colonies with **3c**{*3*,*6*}-impregnated cardboard applicators had significantly fewer dispersing mites than colonies with untreated wooden control applicators, though were still statistically similar to colonies with untreated cardboard control applicators. Differences in Day 84 infestation levels were significant in both AB (*H* = 13.71; df = 4; *P* = 0.009) and BC (*H* = 10.26; df = 4; *P* = 0.036), with the untreated wooden controls in both AB and BC having significantly higher infestations than the Thymovar-treated colonies, and no other significant pairwise comparisons.

Day 0 capped brood infestations in 2022 were similar across treatment groups in both AB (*H* = 0.61; df = 4; *P* = 0.962) (Fig. 2b) and BC (*H* = 1.08; df = 4; *P* = 0.897) (Fig. 2d). Overall Day 0 capped brood infestations were 6.7 ± 1.1% in AB and 3.8 ± 0.6% in BC, across all colonies. Brood infestations differed in AB on Day 35 (*H* = 22.67; df = 4; *P* < 0.001), with the untreated negative control wooden and cardboard groups having significantly higher brood infestations than either **3c**{*3*,*6*}-wood, **3c**{*3*,*6*}-cardboard or Thymovar-treated groups (Supplementary Table S2). Day 42 brood infestations also differed among treatment groups in BC (*H* = 15.30; df = 4; *P* = 0.004), where Thymovar-treated colonies exhibited significantly lower mite infestation levels than the untreated wooden control applicators, with no other significant pairwise comparisons.

### Mortality-based efficacies of **3c***{3*,*6*} treatment

**Fig. 3:**
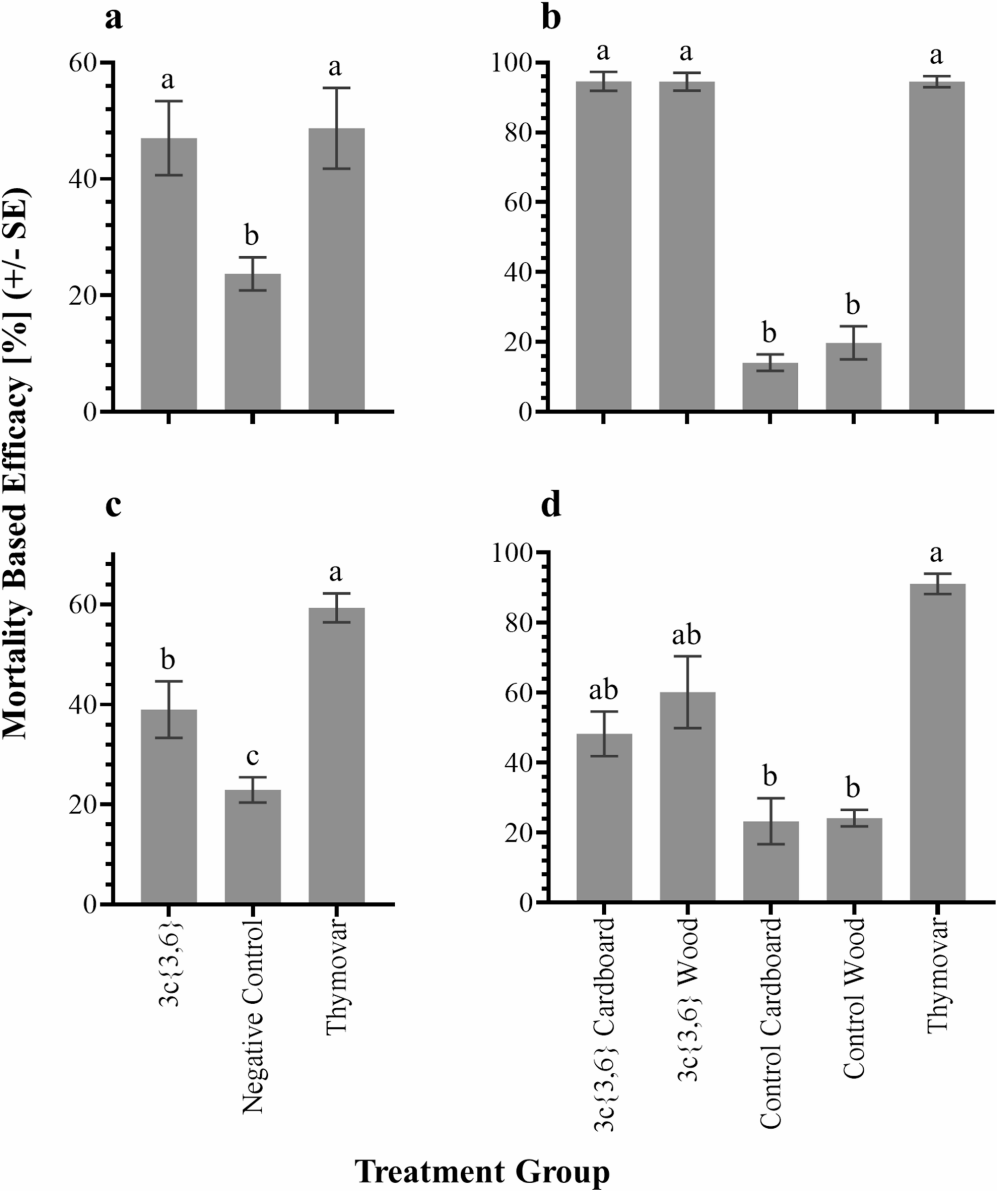
Varroa mite (*Varroa destructor*) mortality-based efficacies of different treatments in the (**a**) AB 2021, (**b**) AB 2022, (**c**) BC 2021, and (**d**) BC 2022 experiments. Values represent mean ± SE. Different letters denote significant post-hoc pairwise differences following 2-way ANOVA and Tukey’s HSD (**c**), or Kruskal-Wallis and Dunn’s post-hoc (**a**,**b**,**d**) tests (α = 0.05). AB = Alberta; BC = British Columbia.

Mortality-based efficacies in the 2021 experiments differed significantly between treatment groups in both AB (*H* = 10.36; df = 2; *P* = 0.006) and BC (*F* = 20.35; df = 2, 27; *P* < 0.001). In AB, **3c**{*3*,*6*} and Thymovar had statistically similar levels of efficacy (42.3 ± 6.6% and 51.2 ± 7.2%, respectively), both being significantly greater than the untreated negative control colonies (23.7 ± 2.8%) (Fig. 3a). Meanwhile, **3c**{*3*,*6*} treatments in BC exhibited significantly lower efficacy (39.7 ± 5.8%) than Thymovar (59.5 ± 2.9%), though both varroacides exhibited higher efficacy than the untreated negative controls (23.3 ± 2.6%) (Fig. 3c).

In 2022, differences in efficacies among treatments were observed in both AB (*H* = 28.34; df = 4; *P* < 0.001) and BC (*H* = 21.05; df = 4; *P* < 0.001). In AB, treatment efficacy was much higher than in 2021. Efficacies for the **3c**{*3*,*6*}-wood (94.5 ± 2.6%) and **3c**{*3*,*6*}-cardboard applicators (94.6 ± 2.7%) were similar to one another, and to Thymovar (94.5 ± 1.6%), with both **3c**{*3*,*6*} treatments being significantly greater than the untreated wooden and cardboard controls (19.8 ± 4.7% and 14.1 ± 2.4%, respectively) (Fig. 3b). In BC, the Thymovar group exhibited the highest efficacy (91.1 ± 2.9%), followed by **3c**{*3*,*6*}-wood (60.2 ± 10.2%) and **3c**{*3*,*6*}-cardboard applicators (48.3 ± 6.4%), with untreated wooden and cardboard controls exhibiting the lowest efficacies (24.2 ± 2.3% and 23.3 ± 6.6%, respectively) (Fig. 3d). Efficacies of Thymovar and both **3c**{*3*,*6*} treatments were statistically similar, as were the two untreated negative controls with one another; untreated controls were also similar to **3c**{*3*,*6*} treatments while Thymovar efficacy was greater than both untreated controls.

*Impact of* **3c***{3*,*6} treatment on daily varroa mortality*.

**Fig. 4:**
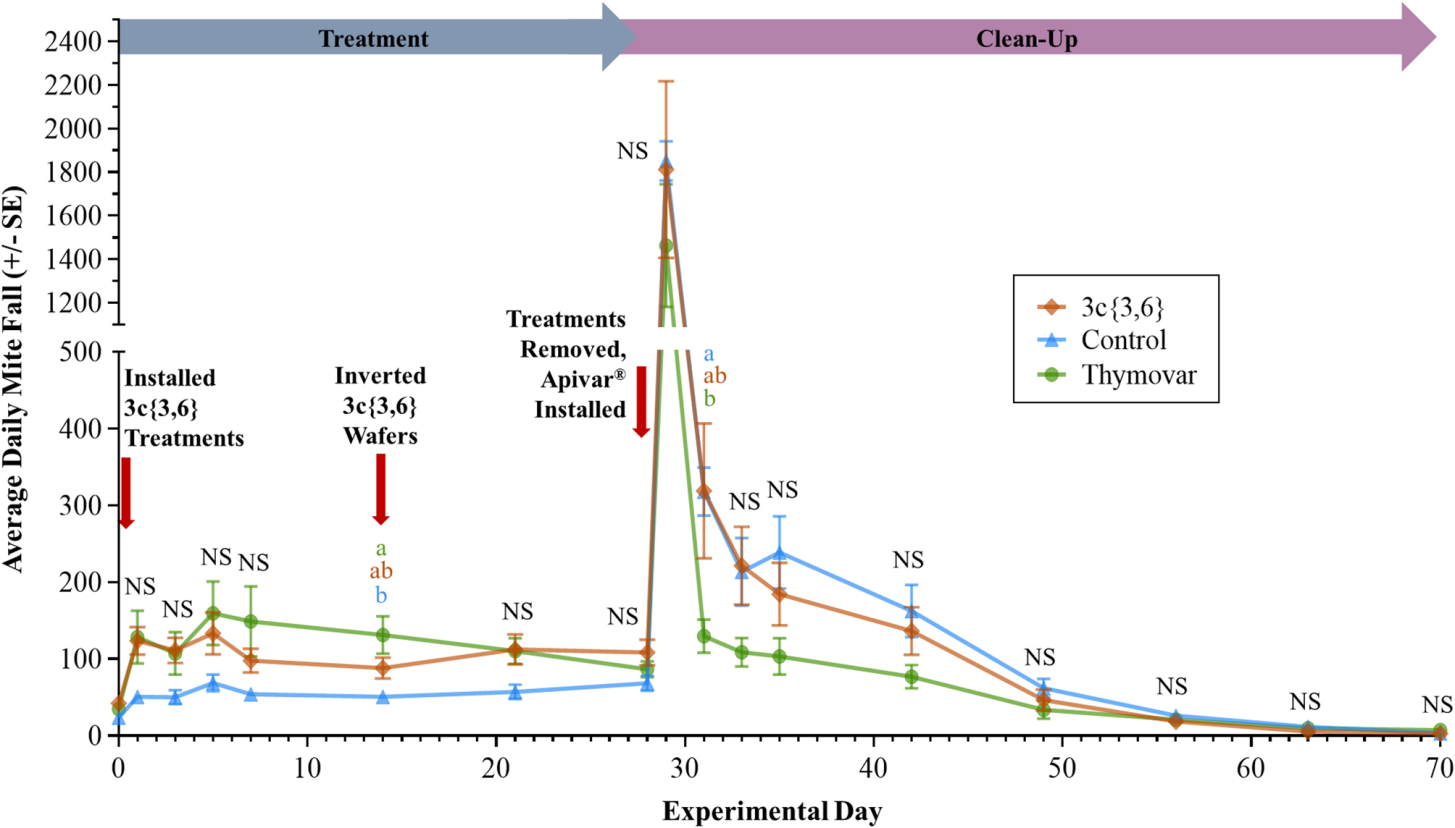
Daily varroa mite (*Varroa destructor*) mortality per sticky board interval in the fall 2021 experiment in Beaverlodge, Alberta, separated by treatment (*n* = 10 per treatment group). Data spans from Day 0 (3 September 2021) to Day 70 (12 November 2021). Points and error bars represent mean ± SE. Three-way mortality comparisons were calculated using a generalized least squares model with global *P*-value adjustments using the Benjamini-Hochberg method and a 5% false discovery rate. Different letters denote significant pairwise differences (α < 0.05, NS = not significant).

**Fig. 5:**
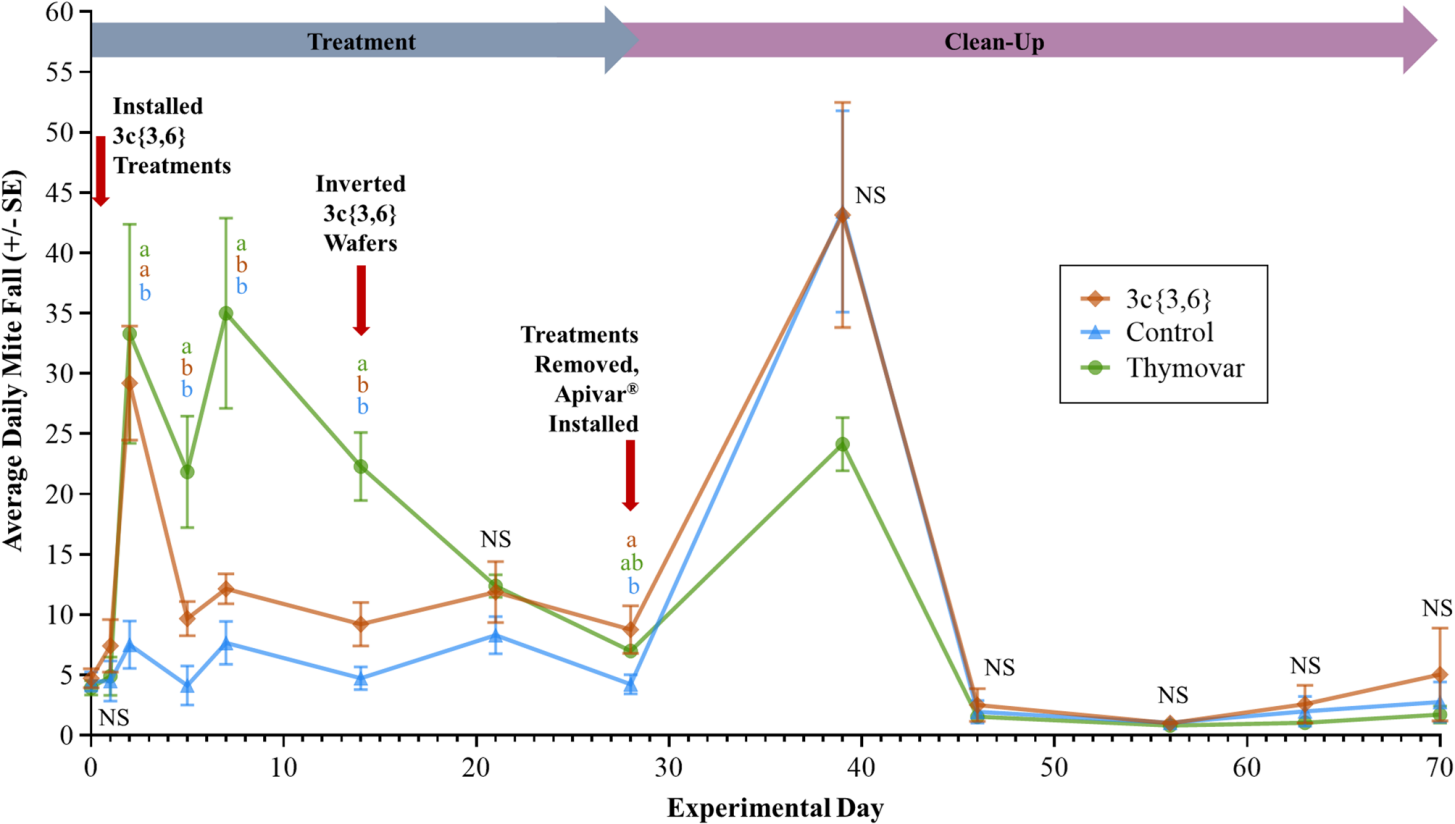
Daily varroa mite (*Varroa destructor*) mortality per sticky board interval in the fall 2021 experiment in Surrey, British Columbia, separated by treatment (*n* = 10 per treatment group). Data spans from Day 0 (1 September 2021) to Day 70 (10 November 2021). Points and error bars represent mean ± SE. Three-way mortality comparisons were calculated using a generalized least squares model with global *P*-value adjustments using the Benjamini-Hochberg method and a 5% false discovery rate. Different letters denote significant pairwise differences (α < 0.05, NS = not significant).

GLS analysis of daily varroa mite fall in the 2021 experiments revealed significant interactions between Treatment and Day main effects for both AB (*F* = 3.69; df = 32, 432; *P* < 0.001) and BC (*F* = 3.97; df = 22, 324; *P* < 0.001). In AB, **3c**{*3*,*6*} and Thymovar-treated colonies exhibited higher mite fall than untreated negative control colonies during the treatment phase, while untreated control colonies had higher mite fall than **3c**{*3*,*6*} and Thymovar-treated colonies during the clean-up phase (Fig. 4), though these differences only reached significance on Days 14 (17 September) and 31 (4 October). Meanwhile, **3c**{*3*,*6*} and Thymovar-treated colonies in BC exhibited a significant spike in varroa mortality on Day 3 (4 September), and Thymovar-treated colonies maintained significantly higher mortality than untreated negative control colonies until Day 21 (22 September) (Fig. 5). It should be noted that severe inclement weather in BC^55,56^ disrupted sticky board exchanges at the start of the clean-up phase, leading to fewer (and longer) monitoring intervals than originally planned. Mite fall levels in all three BC treatment groups showed a strong spike after the initial 11-day interval following the installation of Apivar on Day 39 (10 October), though this trend disappeared by Day 46 (17 October). Nonetheless, in both locations in 2021, mite fall reached its highest level at the start of the clean-up phase for all treatment groups except Thymovar in BC, where the initial mite fall spike in the treatment phase was comparable to the clean-up phase.

**Fig. 6:**
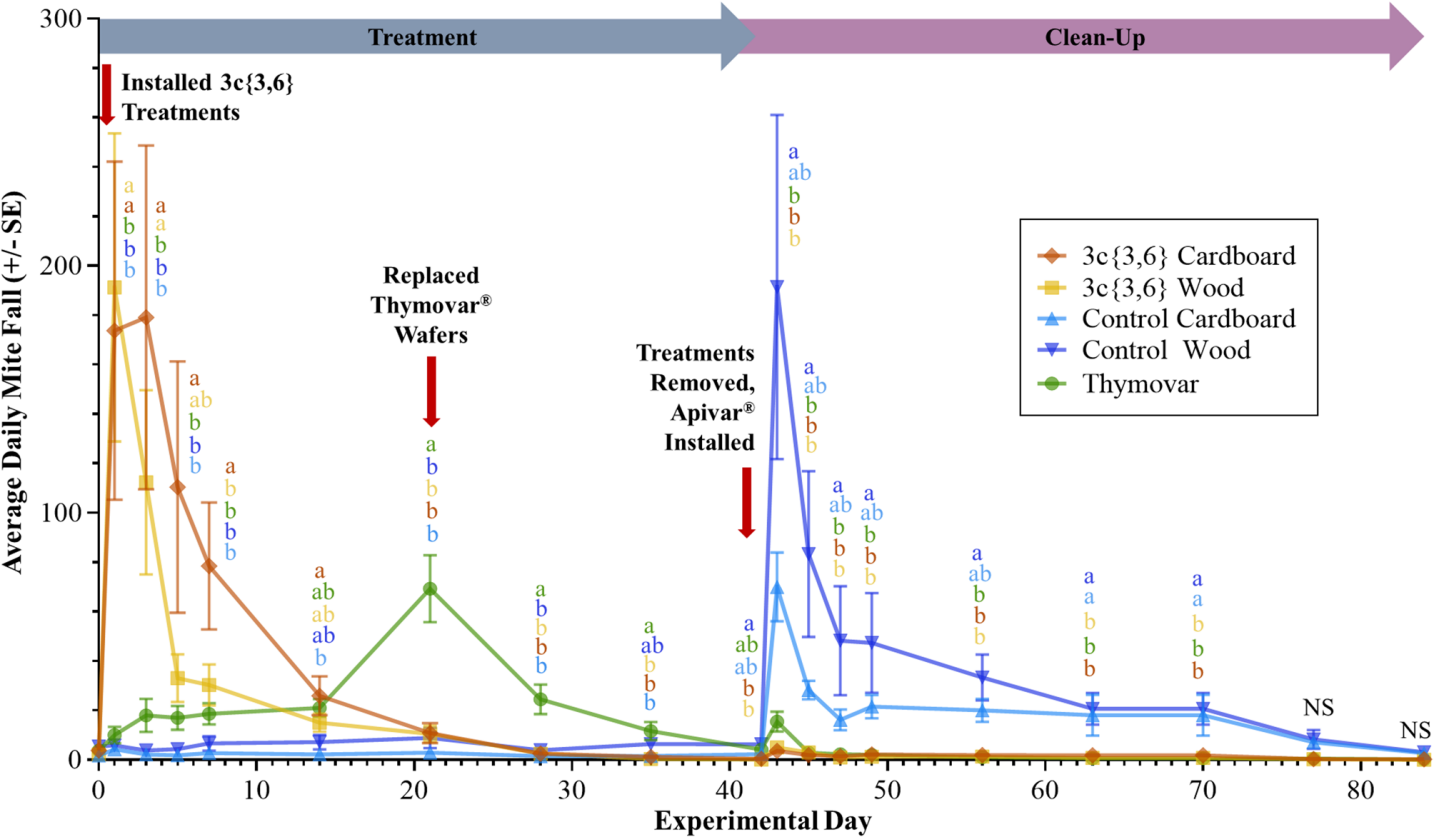
Daily varroa mite (*Varroa destructor*) mortality per sticky board interval in the fall 2022 experiment in Beaverlodge, Alberta, separated by treatment (*n* = 8 per treatment group). Data spans from Day 0 (13 September 2022) to Day 84 (6 December 2022). Points and error bars represent mean ± SE. Five-way mortality comparisons were calculated using a Generalized Least Squares model with global *P*-value adjustments using the Benjamini-Hochberg method and a 5% false discovery rate. Different letters denote significant pairwise differences (α < 0.05, NS = not significant).

**Fig. 7:**
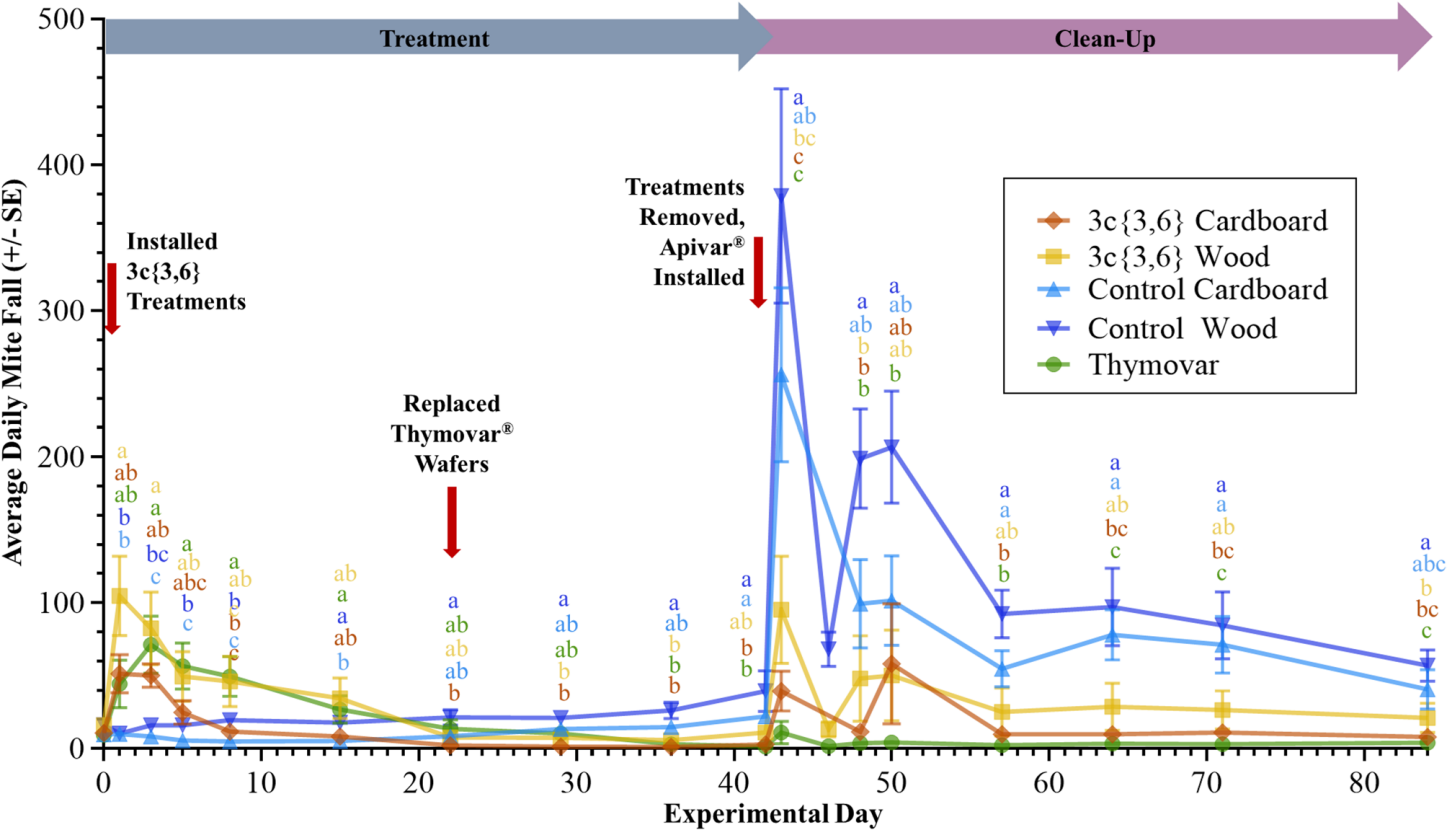
Daily varroa mite (*Varroa destructor*) mortality per sticky board interval in the fall 2022 experiment in the Surrey, British Columbia, separated by treatment (*n* = 8 per treatment group). Data spans from Day 0 (15 July 2022) to Day 84 (6 October 2022). Points and error bars represent mean ± SE. Five-way mortality comparisons were calculated using a Generalized Least Squares model with global *P*-value adjustments using the Benjamini-Hochberg method and a 5% false discovery rate. Different letters denote significant pairwise differences (α < 0.05, NS = not significant).

The 2022 experimental GLS models again showed significant interaction between Treatment and Day main effects in AB (*F* = 2.49; df = 72, 665; *P* < 0.001) and BC (*F* = 2.82; df = 64, 553; *P* < 0.001). In AB, untreated negative control colonies featured consistently low mite fall through the treatment phase (Fig. 6). Meanwhile, colonies treated with both **3c**{*3*,*6*}-wood and **3c**{*3*,*6*}-cardboard applicators showed a significant mite fall peak from the start of the treatment phase on Day 0 until Day 7 (20 September), before dropping down to levels comparable to the untreated controls. Mite fall rates in Thymovar-treated colonies slowly increased until Day 21 (4 October) when the Thymovar group had significantly higher mite fall than all other groups, before this trend subsequently disappeared. Entering the clean-up phase, both untreated control wooden and cardboard groups exhibited noticeable spikes in mortality on Day 43 (26 October), though untreated wooden controls had significantly higher mortality than either **3c**{*3*,*6*} or Thymovar-treated colonies. Both untreated negative controls maintained significantly elevated mite fall until Day 70 before equalizing with the two **3c**{*3*,*6*} and Thymovar cohorts, which all maintained low mite mortality across the entire clean-up phase. BC colonies exhibited a similar pattern, whereby **3c**{*3*,*6*}-treated colonies exhibited a strong mortality spike in the first week of the treatment phase, and both untreated negative control groups featured high mite fall throughout the entire clean-up phase (Fig. 7). However, only colonies with wooden **3c**{*3*,*6*} applicators had significantly higher mite fall versus the untreated controls during the clean-up phase, and only until Day 3 (18 July). Moreover, both untreated negative controls exhibited significantly higher mite fall than the two **3c**{*3*,*6*}-treated cohorts towards the end of the treatment phase, on Day 42. Thymovar-treated colonies also exhibited a mite fall peak at the start of the treatment phase, though it was not significant when compared against either untreated negative control.

### Impact of **3c***{3*,*6*} treatment on food stores, brood, or worker bee populations

All 2021 brood, food, and worker bee measures were similar across treatment groups in AB on Day 0, with the exception of honey stores (*H* = 9.60; df = 2; *P* = 0.008), with **3c**{*3*,*6*}-treated colonies (3.0 ± 0.7 frame sides) having significantly less honey than untreated negative control colonies (6.5 ± 1.0 frame sides), though neither group had significantly different honey stores versus Thymovar-treated colonies (3.8 ± 0.7 frame sides) (Table 1). In BC, all Day 0 colony metrics were similar as well. On Day 28, no colony metrics differed significantly in AB or BC.

**Table 1:** Measurements of honey bee (*Apis mellifera*) colony metrics of experimental colonies throughout the fall 2021 experiments in Alberta and British Columbia. Metrics include areas of capped and open brood, pollen, honey, and adult bees. All measurements were taken on day 0 and day 28. All data are reported as mean ± SE (number of frame sides). Different superscript letters denote significant post-hoc pairwise comparisons (α < 0.05; see Supplementary Table S1).

Location	Experiment Duration	Treatment	Capped Brood (Frame Sides)		Open Brood (Frame Sides)		Pollen (Frame Sides)		Honey (Frame Sides)		Adult Bees (Frame Sides)	
			Day 0	Day 28	Day 0	Day 28	Day 0	Day 28	Day 0	Day 28	Day 0	Day 28
Beaverlodge, AB	3 Sep − 12 Nov (70 days)	**3c**{*3*,*6*}	2.7 ± 0.30	0.8 ± 0.23	0.9 ± 0.17	0.2 ± 0.14	2.0 ± 0.33	2.1 ± 0.38	3.0 ± 0.75^b^	3.3 ± 0.42	13.8 ± 1.33	6.8 ± 0.68
		Untreated negative control	1.9 ± 0.24	0.9 ± 0.20	0.7 ± 0.18	0.3 ± 0.11	1.9 ± 0.42	1.9 ± 0.37	6.5 ± 0.98^a^	3.8 ± 0.49	13.8 ± 0.99	8.1 ± 0.91
		Thymovar	2.4 ± 0.20	0.6 ± 0.14	0.9 ± 0.12	0.2 ± 0.04	1.7 ± 0.34	2.1 ± 0.41	3.8 ± 0.73^ab^	3.7 ± 0.66	14.2 ± 0.72	6.4 ± 0.61
Surrey, BC	1 Sep − 10 Nov (70 days)	**3c**{*3*,*6*}	1.2 ± 0.25	0.0 ± 0.04	1.2 ± 0.32	0.0 ± 0.00	0.6 ± 0.20	0.3 ± 0.09	5.0 ± 0.41	9.4 ± 0.06	n/a	n/a
		Untreated negative control	1.3 ± 0.30	0.1 ± 0.05	0.8 ± 0.18	0.1 ± 0.05	1.6 ± 0.89	0.4 ± 0.11	6.1 ± 0.65	8.6 ± 1.15	n/a	n/a
		Thymovar	1.4 ± 0.21	0.1 ± 0.05	0.8 ± 0.15	0.1 ± 0.05	0.6 ± 0.17	0.2 ± 0.04	4.9 ± 0.65	10.1 ± 0.97	n/a	n/a

n/a – These data were not collected in BC.

Measurements of colonies included in the 2022 experiments are listed in Table 2. Day 0 colony metrics for brood, food, and worker bees did not statistically differ across treatment groups in 2022, for both AB and BC (Supplementary Table S2). On Day 35, AB groups differed in their areas of open brood (*H* = 10.44; df = 4; *P* = 0.034), though no post-hoc pairwise comparisons were significant. BC treatment groups differed in capped brood area on Day 42 (*F* = 3.90; df = 4, 30; *p* = 0.012), with Thymovar-treated colonies having significantly less capped brood than the untreated wooden control group, though no other pairwise comparisons were significant.

**Table 2:** Measurements of honey bee (*Apis mellifera*) colony metrics of experimental colonies throughout the fall 2022 experiments in Alberta and British Columbia. Metrics include areas of capped and open brood, pollen, honey, and adult bees. All measurements were taken on day 0, and day 35 (AB) or day 42 (BC). All data are reported as mean ± SE (number of frame sides). Different superscript letters denote significant post-hoc pairwise comparisons (α < 0.05; see Spplementary Table S2).

Location	Experiment duration	Treatment	Capped brood (frame sides)		Open brood (frame sides)		Pollen (frame sides)		Honey (frame sides)		Adult bees (frame sides)	
			Day 0	Day 35/42	Day 0	Day 35/42	Day 0	Day 35/42	Day 0	Day 35/42	Day 0	Day 35/42
Beaverlodge, AB	13 Sep − 6 Dec (84 days)	**3c**{*3*,*6*} Cardboard	2.9 ± 0.51	1.0 ± 0.18	0.5 ± 0.17	0.2 ± 0.12	2.8 ± 0.75	2.2 ± 0.37	7.0 ± 0.88	14.1 ± 0.63	16.7 ± 0.64	9.5 ± 0.67
		**3c**{*3*,*6*} Wood	2.5 ± 0.31	0.9 ± 0.29	0.6 ± 0.15	0.1 ± 0.04	2.3 ± 0.84	1.7 ± 0.74	7.4 ± 0.90	14.1 ± 0.54	17.0 ± 0.92	8.1 ± 0.34
		Untreated Control Cardboard	3.2 ± 0.44	1.0 ± 0.11	0.4 ± 0.15	0.1 ± 0.07	0.9 ± 0.53	2.3 ± 0.74	8.5 ± 1.15	13.5 ± 0.83	16.4 ± 1.14	8.9 ± 0.60
		Untreated Control Wood	3.1 ± 0.33	1.0 ± 0.18	0.9 ± 0.24	0.4 ± 0.14	2.6 ± 0.80	2.0 ± 0.75	5.8 ± 0.94	13.5 ± 0.88	16.1 ± 0.86	8.7 ± 0.79
		Thymovar	2.9 ± 0.45	0.8 ± 0.16	0.4 ± 0.09	0.3 ± 0.08	2.4 ± 0.90	2.1 ± 0.56	7.7 ± 0.78	13.6 ± 0.84	17.9 ± 2.11	9.4 ± 0.77
Surrey, BC	15 Jul − 6 Oct (84 days)	**3c**{*3*,*6*} Cardboard	2.6 ± 0.57	1.3 ± 0.24^ab^	0.9 ± 0.22	0.5 ± 0.10	0.9 ± 0.18	0.9 ± 0.28	3.3 ± 0.55	5.1 ± 0.45	n/a	n/a
		**3c**{*3*,*6*} Wood	2.8 ± 0.38	1.3 ± 0.42^ab^	1.4 ± 0.38	0.5 ± 0.16	0.9 ± 0.15	1.1 ± 0.41	3.9 ± 0.75	5.1 ± 1.28	n/a	n/a
		Untreated Control Cardboard	3.2 ± 0.48	1.8 ± 0.20^ab^	1.1 ± 0.50	0.5 ± 0.14	1.2 ± 0.33	0.8 ± 0.31	3.5 ± 0.66	4.4 ± 0.72	n/a	n/a
		Untreated Control Wood	3.5 ± 0.51	2.4 ± 0.33^a^	1.1 ± 0.29	1.0 ± 0.23	1.4 ± 0.28	0.8 ± 0.18	4.0 ± 0.78	5.5 ± 1.03	n/a	n/a
		Thymovar	2.4 ± 0.55	1.3 ± 0.66^b^	0.6 ± 0.24	1.0 ± 0.37	0.8 ± 0.13	0.8 ± 0.08	3.4 ± 0.43	4.4 ± 1.24	n/a	n/a

n/a – These data were not collected in BC.

### Fall treatment with **3c***{3*,*6*} and winter survivorship

In the 2021 experimental cohort, three AB colonies died before Day 70 (two **3c**({*3*,*6*}-treated, one Thymovar-treated), while all BC colonies survived. AB pre-winter cluster sizes (2.6 ± 0.3 frame spaces across all colonies) were similar irrespective of treatment (*H* = 0.65; df = 2; *P* = 0.721). BC pre-winter clusters (9.2 ± 0.2 frame spaces across all colonies) were also similar (*H* = 1.09; df = 2; *P* = 0.579). AB overwintering mortality was 90%, with only 3/30 colonies surviving into spring 2022 (one untreated negative control, two Thymovar-treated colonies). In BC, 28/30 colonies survived to spring 2022, with one untreated negative control and one Thymovar-treated colony having died.

In the 2022 experiments, all AB colonies survived to Day 84, with similar pre-winter clusters (2.7 ± 0.3 frame spaces across all colonies) among treatments (*H* = 4.28; df = 4; *P* = 0.368). In BC, six colonies died by Day 84 (one untreated control wood, one **3c**{*3*,*6*}-treated wood, one **3c**{*3*,*6*}-treated cardboard, three Thymovar-treated colonies). In AB, 35/40 colonies survived into spring 2023, with the five dead colonies being comprised of two untreated control wood, one **3c**{*3*,*6*}-treated wood, one **3c**{*3*,*6*}-treated cardboard, and one Thymovar-treated colony. In BC, 19/40 colonies survived wintering (three untreated control wood, four **3c**{*3*,*6*}-treated wood, seven **3c**{*3*,*6*}-treated cardboard, five Thymovar-treated colonies). While a similar number of colonies died per treatment in AB (Fisher’s exact test; *P* = 1.000), colony mortality differed between treatments in BC (Fisher’s exact test; *P* = 0.129), with significantly more **3c**{*3*,*6*}-treated colonies surviving into spring than untreated negative control colonies.

## Discussion

Here we describe the efficacy of **3c**{*3*,*6*} as a varroacide within hive environments. Colonies treated with **3c**{*3*,*6*} during the fall treatment window consistently exhibited elevated daily mite mortality versus untreated control colonies, and were comparable to the Thymovar positive controls, though these differences were not always statistically significant. Mortality-based efficacy of **3c**{*3*,*6*} treatments was also comparable to Thymovar across regions and years. Colonies treated with **3c**{*3*,*6*} did not experience major perturbations in populations of worker bees or brood, nor their honey or pollen stores.

The magnitude of varroacidal effects varied strongly across experiments, in terms of both mortality-based efficacy and post-treatment mite infestation levels. Across all experiments, efficacy of **3c**{*3*,*6*} treatments were comparable to Thymovar and to prior trials, reported in 2023 (51.2% in British Columbia, Canada, and 81.1% in Alberta, Canada)^50^, and 2024 (72.8% in Maryland, USA)^57^. Moreover, both wooden and cardboard applicators in 2022 had comparable efficacies to other commercial varroacide formulations based on thymol, *tau*-fluvalinate, flumethrin, amitraz, and hop organic acids reported across Canada and the United States^58–63^. One potential shortcoming of Dietemann’s formula in our experiment is the assumption that Apivar is close to 100% effective at killing all remaining mites during the clean-up phase, and violation of this assumption would result in an overestimation of efficacy. However, as the final mite washes indicated Apivar to be highly effective at eliminating mites to undetectable levels across all experiments, efficacy overestimation was unlikely. Moreover, while varroa elimination rates between **3c**{*3*,*6*} and Thymovar treatments and untreated negative controls did not always differ significantly, this may be attributable to low baseline varroa infestations, leading to decreased detection power – thus, insignificant differences should be taken with caution. Nonetheless, the baseline infestation rates used in our studies reflect real-world apicultural practices.

We observed that **3c**{*3*,*6*} varroacidal efficacy was highest in 2022 in both AB and BC experimental settings. Several factors may have contributed to the variability of efficacy across experiments, including the increased dosage (8 g in 2022 vs. 4 g in 2021), longer treatment window (6 weeks in 2022, 4 weeks in 2021), and applicator design. The fact that variations in **3c**{*3*,*6*} efficacy coincided with changes in Thymovar efficacy (with Thymovar also showing lower efficacy in 2021 than 2022) supports the importance of treatment duration on efficacy. However, plots of daily mortality versus experimental day show most mite mortality to occur within the days immediately after installation of applicators in 2022, a pattern not as apparent in the 2021 experiments. Moreover, increased Thymovar efficacy in 2022 may also be explained by the replacement of the first strip with a fresh treatment strip on experimental Day 21, enabling a longer period of vapour release within the colonies. Thus, future experiments aiming to optimize **3c**{*3*,*6*} treatment regimens should seek to independently evaluate modifications to each factor, as their individual impacts on efficacy were difficult to clearly separate in our experiments.

Capped brood infestation levels of varroa also varied across years. In 2021, treatment groups in AB showed similar levels of post-treatment brood infestation, with these data being not being measurable in BC because of a lack of brood. In 2022, all **3c**{*3*,*6*} and Thymovar-treated groups in AB had significantly fewer infested brood cells than untreated controls, while in BC only the Thymovar-treated group had statistically lower brood infestations than the untreated wooden control. It is unknown whether reduced brood infestation in **3c**{*3*,*6*}-treated colonies is due to the overall decline of mite populations within hives, or from **3c**{*3*,*6*} vapours penetrating wax caps and acting upon reproductive-stage varroa. One proposed mechanism for reduced brood infestation is the decrease dispersing mite population leading to fewer foundress mites entering brood cells^50^. While it remains unclear whether **3c**{*3*,*6*} vapours can penetrate wax caps, the fact that virtually all reproductive-stage foundresses found during assessments were alive within the **3c**{*3*,*6*}-treated colonies suggests this is unlikely. Impacts on mite reproduction, such as reduced fecundity, could also be possible through **3c**{*3*,*6*} exposure through fumigation of open brood cells, as well as exposure during the dispersal stage. Future experiments should evaluate the specific impact of **3c**{*3*,*6*} on reproductive-stage mites, including the permeability rate of **3c**{*3*,*6*} vapours through capping pores^64^.

Treatment with **3c**{*3*,*6*} did not generally improve overwintering survivorship, with the exception of the 2022 BC experiments, which had higher survival of **3c**{*3*,*6*}-treated colonies than untreated negative control colonies. The 2021 AB experiments were also noteworthy for the high colony loss rates, irrespective of treatment group. The most likely cause of this stems from the very high initial varroa infestation levels at the start of the treatment season. These results suggest that **3c**{*3*,*6*} treatment alone is insufficient to alter poor overwintering prognoses at high infestation levels. Colonies in AB and BC received different prophylactic treatments pre-wintering (fumagillin dicyclohexylamine and HiveAlive supplement respectively), and were overwintered in different conditions (indoors versus outdoors respectively) which may have impacted survivorship rates between locations. However, this does not influence inferences of treatment on overwintering survival.

All colonies experienced a decline in their area of brood and numbers of worker bees from the start of the treatment phase to the end of the experiments. Nevertheless, this observation is likely independent of varroacide treatment and is instead consistent with the shift in colony demographics entering winter^65,66^. Where measured, post-treatment adult bee populations did not differ between colonies treated with **3c**{*3*,*6*} compared to Thymovar-treated or untreated colonies. Generally, varroacide treatment also did not impact brood populations. Though a significant treatment difference was found in the analysis of Day 35 open brood populations for AB in 2022, no significant pairwise differences were detected in subsequent post-hoc analyses. Colonies from BC in 2022 differed in their post-treatment capped brood areas, whereby untreated wooden control colonies had significantly more brood than Thymovar-treated colonies, with no other significant pairwise differences between groups. Taken together, we did not detect any meaningful impact of **3c**{*3*,*6*} on brood nor adult worker bee populations within colonies at our treatment doses, with most demographic shifts resulting instead from seasonal changes, consistent with previous findings^57,67^.

Treatment with **3c**{*3*,*6*} did not impact food storage, as all treatment groups across all experiments showed similar levels of pollen and honey following the end of the treatment phase. This is generally consistent with observations made by Dawdani et al. (2020)^67^ and Cook et al. (2024)^57^, though the latter study noted increased pollen provisioning in the untreated negative control colonies which we did not observe. Moreover, we observed a general trend among all treatments of increased honey provisioning over time. This may be explained by our treatment regimens occurring during a period of low nectar flow, wherein our colonies were supplementarily fed sugar syrup in preparation for winter. Nevertheless, the potential effect of **3c**{*3*,*6*} in situ treatment on honey bee foraging is a pertinent topic for future investigations, should spring treatment regimens be employed in the future – especially given the historic utility of **3c**{*3*,*6*} as an insect feeding deterrent^51,54,68^. Moreover, the ability of **3c**{*3*,*6*} to sequester into hive products remains unclear, and further exploration is warranted.

While our study indicates **3c**{*3*,*6*} more effectively controls varroa at a per-hive dose of 8 g over 4 g, we did not find a significant effect of applicator material, with both wood and cardboard performing comparably. Worker bees visibly degraded the cardboard material throughout the treatment phase in all colonies equipped with cardboard applicators (Supplementary Fig. S2d), though the impact of this degradation on **3c**{*3*,*6*} varroacidal efficacy appears negligible. Nevertheless, based on the need for a controlled release of **3c**{*3*,*6*} over an extended period, wood is more suitable for the design of future applicators to avoid potential uncontrolled release of the compound through the bee-mediated destruction of cardboard, and to more effectively remove of any active material still present on the applicator material at the end of the treatment period.

In general, our experiments demonstrate sustained varroacidal effect of **3c**{*3*,*6*} throughout the treatment phase when applied to honey bee colonies in situ. Our findings indicate efficacy was strongly impacted by treatment duration and dosage, with 8 g/colony over 42 days having increased efficacy versus 4 g/colony over 28 days. Change in efficacy over time was particularly noticeable in the 2022 experiments, where mortality was primarily concentrated in the days immediately following installation of **3c**{*3*,*6*} applicators. Sustained daily mortality of **3c**{*3*,*6*}-treated colonies was also observed during portions of all experiments, demonstrating consistent effects across different geographic regions and years. Our findings help confirm the viability of **3c**{*3*,*6*} as an effective commercial varroacide and contribute to the optimization of a treatment regimen. Further investigation will be needed to disentangle the specific effects of weather/climate, dosage, and applicator design.

Additional exploration should also be made to better understand the impacts of **3c**{*3*,*6*}exposure on honey bee and human health, particularly for beekeepers exposed to levels of **3c**{*3*,*6*} used to treat honey bee colonies. Until exposure assessments are available, a minimum PPE requirement of chemical-resistant gloves, long sleeves and eye protection should be used, and procedures for safe handling of organic solvents followed.

## Materials and methods

### Colony management and selection

Parallel experiments in 2021 and 2022 were performed in two locations in Canada: at Agriculture and Agri-Food Canada’s Research Farm in Beaverlodge, Alberta (AB), and in Surrey, British Columbia (BC). Experimental colonies were established by splitting local colonies where possible, otherwise they were constituted from imported package bees. Across all experiments, colonies were managed in standard-sized Langstroth deep hive boxes with nine frames. During the honey flow (July to August in AB, May to July in BC), colonies were equipped with a two Langstroth deep honey supers, each with nine frames (AB), or one medium depth honey super with ten frames (BC), separated from the broodnest using a queen excluder. Colonies were grouped atop wooden pallets in groups of two to four.

Dispersal-stage mite infestation levels were evaluated in all colonies using the alcohol wash method, whereby ~ 300 worker bees were collected from the central broodnest and loaded into a mesh-bottomed container nested within a solid-bottomed container^69,70^. Bees were submerged in 70% ethanol (AB) or 50% isopropanol and sealed, before being agitated for three cycles of five, five and ten minutes using an orbital shaker (MBIMS-NOR-30, Montréal Biotech Inc., Montréal, QC) (AB) or by hand (BC) to dislodge mites. The number of dislodged mites were counted from the solid-bottom container and summed, and a weight regression was used to calculate the total number of bees in the sample (AB)^71^, or were directly counted (BC). Percent dispersing mite infestation was calculated by dividing total dislodged mites by the number of sampled bees, multiplied by 100%. Colonies in AB required a minimum dispersal-stage infestation of 3% to be considered for the experiment^72,73^, while in BC, colonies were included if they had 1–3% mite infestations to account for the longer mite rearing periods in that region and the recommended threshold^30^.

Supplemental pollen patties (Global Patties, Airdrie, AB) containing 15% pollen by weight were provided to colonies as needed in the spring and fall, as a single patty (454 g) split into two halves. During periods of low nectar flow in September (AB) or July (BC), colonies were fitted with hive-top feeders (BeeMaid Bee Supplies, Spruce Grove, AB) or frame feeders (Urban Bee Supplies, Delta, BC) and fed 66% (w/v) sucrose syrup purchased from Lantic Inc. (Taber, AB) (AB), or mixed from table sugar (sucrose crystals) in-house (BC). Following the end of the experiments, colonies were provisioned with 12–16 L syrup, supplemented in AB with 90 mg/colony fumagillin dicyclohexylamine to prevent nosemosis^74^, and with HiveAlive nutrient supplement (Urban Bee Supplies, Delta, BC) in BC.

### General experimental design

The experimental timeline was divided into three distinct phases: setup, treatment, and clean-up^50^. Dates and durations of each phase varied by year and location (detailed later). Experimental colonies were placed atop mesh screen bottom boards (Propolis-etc… [sic], St-Mathieu se Beloeil, Québec), within which varroa mite adhesive (“sticky”) boards (#M00935, Dadant and Sons Inc., Hamilton, IL) were positioned to monitor mite mortality. An initial sticky board was placed at the start of the setup phase and was exchanged with a fresh board at the start of the treatment phase to monitor mite fall activity prior to treatment application. Subsequently, sticky boards were exchanged at 7-day intervals (when possible), except during the first week of both treatment and clean-up phases where the first exchange occurred after a single day, followed by three, two-day exchange intervals. Daily mite mortality was determined by dividing the total mite count per board by the length (in days) of the interval.

During the setup phase, experimental colonies were randomized into treatment groups within stratified mite infestation levels, as determined via alcohol wash. This ensured all treatment groups began with consistent mite infestation levels. Mite infestation levels in capped brood were also evaluated when sufficient pink/purple-eyed pupae was present, by temporarily removing sealed brood frames from each colony and uncapping 100 cells/frame^75,76^. Pupae were extracted from cells and scored as to whether a foundress was present, whether the foundress was alive or dead, and whether mite progeny were present (though very few foundresses were non-reproductive). Several other colony level factors were visually assessed as well, including areas of food stores, brood, and adult bees. Areas of colony food stores and brood were evaluated using 8 × 18 grids (grid square increments = 2.54 cm), while adult bee populations were visually estimated to the nearest quarter-frame side. These measurements were subsequently converted to total frame sides for analyses.

The treatment phase (experimental Day 0) began with an alcohol wash to establish initial varroa infestation levels, alongside the installation of applicators (**3c**{*3*,*6*}, untreated negative control, or Thymovar) into respective treatment group colonies. Another set of colony evaluations (food, brood, bees, mite infestation) were performed at the end of the treatment phase, using the methods described previously. Length of the treatment phase varied by year, lasting 28 days in 2021 and 42 days in 2022.

Entering the clean-up phase, all treatment applicators were removed and Apivar (3.33% amitraz) was placed in all colonies, irrespective of treatment group. At the end of the clean-up phase, a third mite alcohol wash was performed to evaluate final dispersal-stage varroa infestation levels.

### Colony overwintering

Cluster evaluations occurred after fall feeding was completed, immediately prior to wintering. The size of the pre-wintering cluster was visually assessed, viewed from the top of the brood chamber^77^. In 2022, pre-wintering clusters were not evaluated in BC.

In AB, the start of the overwintering period began when hives were moved into an indoor wintering room, where air circulation was provided by a ceiling-mounted fan and temperature controlled at 4–5° C using a combination of a forced-air furnace and a thermostatically-controlled exhaust fan^66,78^. In 2021, colonies were moved indoors at the end of the clean-up phase on 12 November, while in 2022, the clean-up phase was still ongoing when colonies were moved indoors on 31 October. In BC, colonies were wintered outdoors. Here, the wintering period was associated with the end of feeding and winter preparations, which occurred on 16 November 2021 and 21 November 2022. In early spring of the subsequent years, colonies were evaluated for winter survival (presence of both live queen and workers), on 26 April 2022 and 1 May 2023 in AB, and 1 March 2022 and 17 February 2023 in BC.

### Field efficacy experiments of **3c***{3*,*6*} in 2021

The 2021 experiments included 30 colonies per location, distributed into three different treatment groups (*n* = 10 per treatment): **3c**{*3*,*6*}, negative control (untreated), and Thymovar (positive control). AB colonies were established by splitting local hives of mixed Italian and New World Carniolan-stock in mid-May and then re-queening with New World Carniolan queens (Olivarez Honey Bees, Orland, CA), bred in 2021. The parental colonies for producing the splits were not previously treated for varroa mites in the fall of 2020 nor the spring of 2021. In BC, colonies were founded using 1-kg New Zealand package bees containing approximately 10,000 workers (Kintail Honey Ltd., New Zealand). Packages were shipped with a small Apivar strip that was removed upon hiving the package. These bees were installed during mid-April into standard-sized Langstroth deep hive boxes with nine frames, which were subsequently requeened with same source of New World Carniolan stock used for the Beaverlodge colonies. All colonies were equalized to the same nominal size, and natural varroa infestations were sufficient to require treatment by the start of the experiments. In AB, colonies were housed at the “Varroa Yard” (55.30007° N, 119.28215° W), while BC colonies were kept at a bee yard in South Surrey (49.01558 ^o^ N 122.706758 ^o^ W).

Experimental applicators in 2021 were single wooden strips (15.3 cm × 5.1 cm × 0.5 cm) containing 4 g of **3c**{*3*,*6*}, made by dissolving the compound in 40 mL isopropanol with 2% glycerol (v/v) solution and then drizzling it on strips, letting the isopropanol evaporate fully between applications (Supplementary Fig. S1). Both sides of each applicator were coated. Untreated negative control applicators used identical strips, albeit only treated with glycerol-isopropanol carrier solvent. Both **3c**{*3*,*6*} and untreated negative control strips were laid flat over top of the top bars in the centre of the broodnest. Positive control colonies received Thymovar per label instructions, with each strip containing 15 g thymol. Each positive control colony received a single Thymovar strip cut into two halves, with each half placed at opposing corners of the broodnest atop the frames.

The 2021 treatment phase lasted 28 days, beginning on Day 0 (3 September in AB, 1 September in BC) and ending on Day 28 (1 October in AB, 29 September in BC). On Day 14, all Thymovar applicators were inverted per manufacturer label instructions, as well as all **3c**{*3*,*6*} and untreated control applicators for consistency. The 42-day clean-up phase commenced immediately after the removal of applicators on Day 28, and continued until Day 70 (12 November in AB, 10 November in BC). Apivar was applied per label instructions, with two strips per colony placed in the broodnest, hanging between combs. Full colony assessments (food, brood, bees, brood infestation) were performed on Days 0 and 28, while dispersing mite alcohol washes were performed on Days 0, 28, and 70. A full timeline of the fall 2021 AB experiments can be found in Supplementary Table S3, and BC experiments in Supplementary Table S4.

### Field efficacy experiments of **3c***{3*,*6*} in 2022

The 2022 experiments included 40 colonies per location, equally distributed into five treatment groups (*n* = 8). Two **3c**{*3*,*6*} applicator materials were tested: wood and cardboard. Thus, groups were stratified by both treatment compound and applicator material: **3c**{*3*,*6*} (wood and cardboard), untreated negative control (wood and cardboard), and Thymovar (positive control). AB colonies were founded from splits of locally-bred colonies, requeened on 7 June with New World Carniolan queens (Olivarez Honey Bees, Orland, CA). The parental hives from which the splits were derived were not treated with varroacides in the spring of 2022 but were treated with Apivar in the fall of 2021. Consequently, the splits lacked sufficient varroa and required inoculations of mites sourced from other local hives in the Beaverlodge area. Dispersal-stage mites were collected via sugar shake, whereby frames of bees from varroa-infested colonies were shaken into powdered sugar-filled bins and agitated to dislodge mites, which were sieved out via coarse mesh^79^. Mites were then collected, gently wiped clean using a damp fine-tipped paintbrush, and manually placed on nurse bees within experimental colonies. A total of 42 mites were inoculated into each colony between 8 and 14 July 2022, by depositing individual mites onto abdomens of nurse bees within the broodnest using a fine-tipped paint brush. Varroa populations in a small number of AB colonies remained low even post-inoculation and were substituted with local Beaverlodge colonies exhibiting above-threshold infestation levels; these contained locally bred one- or two-year-old queens. BC colonies in 2022 were overwintered from 2021, supplemented with additional 1-kg New Zealand package bees during April. Overwintered colonies had previously been treated for varroa mites in the fall of 2021 with oxalic and formic acid; packages received in the spring of 2022 were shipped with a small Apivar strip which was removed upon hiving. Natural varroa infestations in BC colonies exceeded mite threshold requirements for the experiment. AB colonies were initially housed at the “Anderson Lake” apiary (55.20135° N, 119.15251° W), but were moved on Day 28 (11 October) to the “Home Yard” site (55.20278° N, 119.39644° W); BC colonies were kept at the “South Surrey” apiary (described above).

In 2022, the treatment phase lasted 42 days, spanning from Day 0 (13 September in AB, 15 July in BC) to Day 42 (5 October in AB, 26 August in BC). This was followed by a six-week clean-up with Apivar which lasted until Day 84 (6 December in AB, 6 October in BC). Full colony evaluations were performed on Day 0 in AB and BC, Day 35 in AB (18 October) due to cold weather, and across Days 42 and 43 (25 and 26 August) in BC due to time constraints. A full timeline of the fall 2022 AB experiments can be found in Supplementary Table S5, and BC experiments in Supplementary Table S6.

The **3c**{*3*,*6*} applicators in 2022 consisted of three compound-impregnated strips of wood (24.0 cm × 5.0 cm × 0.5 cm) or corrugated cardboard (24.0 cm × 5.0 cm × 0.3 cm) hung vertically between broodnest frames (Supplementary Fig. S2a-b), supported by a fourth untreated strip of similar size and dimensions laid across the top bars (Fig S2 C). A total of 8 g of **3c**{*3*,*6*}was applied per colony, dispersed evenly among the three hanging strips. To formulate these, 6 g **3c**{*3*,*6*} was first dissolved in 60 mL glycerol-isopropanol solution (2%, v/v) and drizzled on, allowing drying between applications, then a final 2 g was dissolved in 100% isopropanol and applied. This created a faster-releasing outer surface layer dissolved in 100% isopropanol, alongside a slower-release lower layer dissolved in the glycerol-isopropanol mixture. Untreated control applicators received only carrier solvent (both glycerol-isopropanol mix, and 100% isopropanol). Thymovar was again used as a positive control whereby two Thymovar strips were used per colony, with single strips cut into halves and placed at opposing corners on Day 0, before being replaced by new strips on Day 21 (4 October in AB, 5 August in BC).

### Statistical analyses

Data structures of colony metrics, overwintering cluster sizes, and calculated efficacies were evaluated for normality via a Shapiro-Wilk test, and for variance structure using Levene’s test. Comparisons of variables across treatment groups (including food stores, brood and adult worker bee populations, and brood and dispersing mite infestations per sampling timepoint) were made using one-way ANOVA with Tukey’s post-hoc tests where normality and homoscedasticity were met, or else with Kruskal-Wallis and Dunn’s post-hoc tests.

Mortality-based efficacies of data were calculated for each treatment group using Dietemann’s formula as outlined below:

$$\:\%\:Efficacy=\:\frac{{mortality}_{treat}}{{mortality}_{treat}+{mortality}_{clean}}\times\:100\%$$

The term *mortality*_*treat*_ refers to the total number of mites killed during the treatment phase, and *mortality*_*clean*_ is the total number of mites during the clean-up phase, based on sticky board mite count data. In 2021, *mortality*_*treat*_ extended from Day 0 to Day 28, and *mortality*_*clean*_ from Day 28 to Day 70. In 2022, *mortality*_*treat*_ extended from Day 0 to Day 42, and *mortality*_*clean*_ from Day 42 to Day 84. Efficacy was first calculated at the per-colony level, and subsequently were averaged per treatment group for statistical comparisons. Colonies that did not survive until the end of the experiment were excluded from efficacy calculations, and the missing datapoints from the BC 2022 experiment for colonies with cardboard applicators on Day 46, and all colonies on Day 78, were censored from the calculations.

A generalized least squares (GLS) approach was used to model daily mite mortality per treatment over each measurement interval, to determine interactive effects between treatment and time. A first-order autoregression term based on colony ID was incorporated to account for temporal autocorrelation stemming from repeated measurements. Models were constructed in the R statistical environment v4.1.1^80^ with the following formula input into the *gls* function in the “nlme” package v3.1-151^81^:

gls(Mites~Treatment*Day, data=mitefall[year], correlation=corAR1(form=~1|Colony), weights=varIdent(form=~1|Treatment*Day_int), method=“ML”)

Robust variance-covariance estimators were subsequently created for each model with the *vcovVR* function from package “clubSandwich” v0.5.11^82^, to accommodate potential mild deviations from the autoregression model.

The BC 2022 dataset had missing data on Day 46 (29 August) for the **3c**{*3*,*6*}-impregnated and untreated control cardboard applicator treatments, and on Day 78 (30 September) for all treatments. As GLS is incompatible with missing values over variables^83^, Day 46 datapoints were censored for the linear model analysis. However, these data were included in efficacy calculations and the BC 2022 graph (Fig. 7). The Day 78 datapoint was excluded from all analyses. While the exclusion of this datapoint impacts sensitivity of both the GLS models and efficacy calculations aforementioned, as they occurred to all colonies on the same dates (including untreated negative controls), they do not impact the conclusions based upon between-group comparisons.

Throughout the experiments, several colonies variously died during the clean-up phase. These were excluded from efficacy calculations, final mite alcohol washes, and cluster evaluations. In the GLS models, these colonies’ mortality data were censored to the end of the treatment phase.

Pairwise comparisons of per-interval mite mortalities across treatment groups per sticky board were performed with the *glht* function in package “multcomp” v1.4-26^84^. Global *P*-value adjustments were subsequently performed for all pairwise comparisons at all days, using the Benjamini-Hochberg method with a false discovery rate (FDR) of 5%.

Effect of treatment on overwintering survival was evaluated using a two-sided Fisher’s exact test for count data, performed with the *fisher_test* function in package “rstatix” v0.7.2 and a Monte Carlo simulation with 10^6 simulations^85^:

$${\mathrm{fisher}}\_{\mathrm{test}}\left( {{\mathrm{winter}}\_{\mathrm{survival}}\left[ {{\mathrm{year}}} \right],{\text{ simulate}}.{\mathrm{p}}.{\mathrm{value}} = {\mathrm{TRUE}},{\text{ B}} = {\mathrm{1}}000000} \right)$$

Post-hoc pairwise comparisons were subsequently performed where survivorship differed significantly at α = 0.05, using the *pairwise_fisher_test* function^85^, with a Holm-Bonferroni *P*-value adjustment for multiple comparisons. For 2022 data, wooden and cardboard cohorts of the same treatment (**3c**{*3*,*6*} and untreated negative control) were combined, and applicator material was disregarded.

## Supplementary Information

Below is the link to the electronic supplementary material.

Supplementary Material 1

Supplementary Material 2

Supplementary Material 3

## Data Availability

Raw data collected during all experiments can be found in the Supplementary_Data.xlsx file, as a formatted Excel spreadsheet containing the following four sheets: BeaverlodgeData2021, BeaverlodgeData2022, SurreyData2021, and SurreyData2022. Sheets contain the location, year, experimental start and end dates, and dates of treatment installation. Dispersing and brood infestation rates are measured in percent infestation. Mite fall is measured in count data of varroa mites recorded for the entire interval. Measures of brood, food and adult bee populations are recorded in number of frame side surfaces covered. Detailed results of statistical tests used for each colony metric for each experiment can be found in Supplementary Table S1 for the 2021 experiments, and Supplementary Table S2 for the 2022 experiments. Detailed experimental timelines can be found in Supplementary Tables S3 to S6. All supplementary tables can be found in the Supplementary_Tables.docx file. Images of the 2021 and 2022 3c{3,6} applicators can be found in the Supplementary_Figures.docx file.
