# Correction: Field trials of the novel varroacide, 1-allyloxy-4-propoxybenzene, against *Varroa destructor* in Western Canada

**DOI:** 10.1038/s41598-026-61788-w

**Published:** 2026-08-18

**Authors:** Robert X. Lu, Abdullah Ibrahim, Olav Rueppell, Erika Plettner, Stephen F. Pernal

**Affiliations:** 1 Department of Biology, University of Oxford, Oxford, UK; 2 Beaverlodge Research Farm, Agriculture and Agri-Food Canada, Beaverlodge, AB Canada; 3 Department of Biological Sciences, University of Alberta, Edmonton, AB Canada; 4 Department of Chemistry, Simon Fraser University, Surrey, BC Canada

Correction to: *Scientific Reports* 10.1038/s41598-025-23935-7, published online 17 November 2025

The original version of this Article contained an error in the copyright line.

“© Crown 2025”

now reads:

“© His Majesty the King in Right of Canada, as represented by the Minister of Agriculture and Agri-Food Canada, 2025”

The original Article has been corrected.

