# Supplementary material for: Field trials of the novel varroacide, 1-allyloxy-4-propoxybenzene, against Varroa destructor in Western Canada
Source: Sci Rep. 2025 Nov 17;15:40183. doi: 10.1038/s41598-025-23935-7 (PMC12623769; doi:10.1038/s41598-025-23935-7)
Supplement: Supplementary file 2 — Supplementary Material 2 [file 41598_2025_23935_MOESM2_ESM.docx]

**Table S1:** Statistical tests of measured colony metrics between treatment groups in the 2021 experiments. Post-hoc pairwise comparisons performed for metrics where *p*-values < 0.05. Different letters among treatments represent significant pairwise differences (α < 0.05).

| **Location** | **Day** | **Metric** | ***n*** | **Test + Post-Hoc** | **df** | **Statistic** | ***p*-Value** | **Pairwise Differences** | | | | |
| --- | --- | --- | --- | --- | --- | --- | --- | --- | --- | --- | --- | --- |
|  |  |  |  |  |  |  |  | **3c{3,6}** | **Negative Control** | | **Thymovar** | |
| AB | 0 | Alcohol Wash | 30 | One-Way ANOVA + Tukey | 2, 27 | 1.67 | 0.206 | n/a | | | | |
|  |  | Brood Infestation | 30 | One-Way ANOVA + Tukey | 2, 27 | 0.31 | 0.738 |  |  |  |  |  |
|  |  | Capped Brood | 30 | One-Way ANOVA + Tukey | 2, 27 | 2.63 | 0.090 |  |  |  |  |  |
|  |  | Open Brood | 30 | One-Way ANOVA + Tukey | 2, 27 | 0.27 | 0.764 |  |  |  |  |  |
|  |  | Pollen | 30 | One-Way ANOVA + Tukey | 2, 27 | 0.20 | 0.818 |  |  |  |  |  |
|  |  | Honey | 30 | One-Way ANOVA + Tukey | 2, 27 | 4.94 | 0.015 | b | | a | | ab |
|  |  | Adult Bees | 30 | Kruskal-Wallis + Dunn | 2, 27 | 0.05 | 0.973 | n/a | | | | |
|  | 28 | Alcohol Wash | 30 | Kruskal-Wallis + Dunn | 2 | 0.94 | 0.626 | n/a | | | | |
|  |  | Brood Infestation | 30 | One-Way ANOVA + Tukey | 2, 27 | 0.58 | 0.566 |  |  |  |  |  |
|  |  | Capped Brood | 30 | Kruskal-Wallis + Dunn | 2 | 2.17 | 0.338 |  |  |  |  |  |
|  |  | Open Brood | 30 | Kruskal-Wallis + Dunn | 2 | 1.29 | 0.524 |  |  |  |  |  |
|  |  | Pollen | 30 | One-Way ANOVA + Tukey | 2, 27 | 0.06 | 0.942 |  |  |  |  |  |
|  |  | Honey | 30 | One-Way ANOVA + Tukey | 2, 27 | 0.21 | 0.810 |  |  |  |  |  |
|  |  | Adult Bees | 30 | Kruskal-Wallis + Dunn | 2 | 2.25 | 0.325 |  |  |  |  |  |
|  | 70 | Alcohol Wash | 23 | Kruskal-Wallis + Dunn | 2 | 6.33 | 0.042 | b | ab | | a | |
| BC | 0 | Alcohol Wash | 30 | Kruskal-Wallis + Dunn | 2 | 0.11 | 0.946 | n/a | | | | |
|  |  | Capped Brood | 30 | One-Way ANOVA + Tukey | 2, 27 | 0.19 | 0.828 |  |  |  |  |  |
|  |  | Open Brood | 30 | Kruskal-Wallis + Dunn | 2 | 1.06 | 0.587 |  |  |  |  |  |
|  |  | Pollen | 30 | Kruskal-Wallis + Dunn | 2 | 0.08 | 0.960 |  |  |  |  |  |
|  |  | Honey | 30 | One-Way ANOVA + Tukey | 2, 27 | 1.20 | 0.318 |  |  |  |  |  |
|  | 28 | Alcohol Wash | 30 | One-Way ANOVA + Tukey | 2, 27 | 1.24 | 0.307 | n/a | | | | |
|  |  | Capped Brood | 30 | Kruskal-Wallis + Dunn | 2 | 3.72 | 0.156 |  |  |  |  |  |
|  |  | Open Brood | 30 | Kruskal-Wallis + Dunn | 2 | 2.98 | 0.225 |  |  |  |  |  |
|  |  | Pollen | 30 | Kruskal-Wallis + Dunn | 2 | 1.06 | 0.584 |  |  |  |  |  |
|  |  | Honey | 30 | One-Way ANOVA + Tukey | 2, 27 | 0.67 | 0.521 |  |  |  |  |  |
|  | 70 | Alcohol Wash | 30 | Kruskal-Wallis + Dunn | 2 | 2.76 | 0.252 | n/a | | | | |

*n* represents number of colonies included in measurement

**Table S2:** Statistical tests of measured colony metrics between treatment groups in the 2022 experiments. Post-hoc pairwise comparisons performed for metrics where *p*-values < 0.05. Different letters among treatments represent significant pairwise differences (α < 0.05).

| **Location** | **Day** | **Metric** | ***n*** | **Test** | **df** | **Statistic** | ***p*-Value** | **Post-Hoc** | | | | |
| --- | --- | --- | --- | --- | --- | --- | --- | --- | --- | --- | --- | --- |
|  |  |  |  |  |  |  |  | **3c{3,6} Cardboard** | **3c{3,6}**  **Wood** | **Control Cardboard** | **Control Wood** | **Thymovar** |
| AB | 0 | Alcohol Wash | 40 | Kruskal-Wallis + Dunn | 4 | 0.03 | 1.000 | n/a | | | | |
|  |  | Brood Infestation | 40 | Kruskal-Wallis + Dunn | 4 | 0.61 | 0.962 |  |  |  |  |  |
|  |  | Capped Brood | 40 | One-Way ANOVA + Tukey | 4, 35 | 0.40 | 0.806 |  |  |  |  |  |
|  |  | Open Brood | 40 | Kruskal-Wallis + Dunn | 4 | 3.04 | 0.551 |  |  |  |  |  |
|  |  | Pollen | 40 | Kruskal-Wallis + Dunn | 4 | 3.80 | 0.434 |  |  |  |  |  |
|  |  | Honey | 40 | One-Way ANOVA + Tukey | 4, 35 | 1.10 | 0.373 |  |  |  |  |  |
|  |  | Adult Bees | 40 | Kruskal-Wallis + Dunn | 4 | 0.73 | 0.948 |  |  |  |  |  |
|  | 35 | Brood Infestation | 35 | Kruskal-Wallis + Dunn | 4 | 22.67 | <0.001 | b | b | a | a | b |
|  |  | Capped Brood | 40 | Kruskal-Wallis + Dunn | 4 | 1.44 | 0.838 | n/a | | | | |
|  |  | Open Brood | 40 | Kruskal-Wallis + Dunn | 4 | 10.44 | 0.034* |  |  |  |  |  |
|  |  | Pollen | 40 | Kruskal-Wallis + Dunn | 4 | 1.40 | 0.845 |  |  |  |  |  |
|  |  | Honey | 40 | One-Way ANOVA + Tukey | 4, 35 | 0.17 | 0.950 |  |  |  |  |  |
|  |  | Adult Bees | 40 | One-Way ANOVA + Tukey | 4, 35 | 0.78 | 0.545 |  |  |  |  |  |
|  | 42 | Alcohol Wash | 40 | Kruskal-Wallis + Dunn | 4 | 23.85 | <0.001 | bc | c | ab | a | bc |
|  | 84 | Alcohol Wash | 40 | Kruskal-Wallis + Dunn | 4 | 13.71 | 0.008 | ab | ab | ab | a | b |
| BC | 0 | Alcohol Wash | 40 | Kruskal-Wallis + Dunn | 4 | 7.00 | 0.136 | n/a | | | | |
|  |  | Brood Infestation | 40 | Kruskal-Wallis + Dunn | 4 | 1.08 | 0.897 |  |  |  |  |  |
|  |  | Capped Brood | 40 | One-Way ANOVA + Tukey | 4, 35 | 0.71 | 0.588 |  |  |  |  |  |
|  |  | Open Brood | 40 | Kruskal-Wallis + Dunn | 4 | 3.57 | 0.467 |  |  |  |  |  |
|  |  | Pollen | 40 | Kruskal-Wallis + Dunn | 4 | 2.87 | 0.579 |  |  |  |  |  |
|  |  | Honey | 40 | One-Way ANOVA + Tukey | 4, 35 | 0.21 | 0.932 |  |  |  |  |  |
|  | 41-42 | Alcohol Wash | 34 | Kruskal-Wallis + Dunn | 4 | 21.13 | 0.000 | bc | abc | ab | a | c |
|  |  | Brood Infestation | 32 | Kruskal-Wallis + Dunn | 4 | 15.30 | 0.004 | ab | ab | ab | a | b |
|  |  | Capped Brood | 35 | One-Way ANOVA + Tukey | 4, 30 | 3.90 | 0.012 | ab | ab | ab | a | b |
|  |  | Open Brood | 35 | Kruskal-Wallis + Dunn | 4 | 4.14 | 0.388 | n/a | | | | |
|  |  | Pollen | 35 | Kruskal-Wallis + Dunn | 4 | 2.26 | 0.688 |  |  |  |  |  |
|  |  | Honey | 35 | One-Way ANOVA + Tukey | 4, 30 | 1.58 | 0.206 |  |  |  |  |  |
|  | 84 | Alcohol Wash | 31 | Kruskal-Wallis + Dunn | 4 | 10.26 | 0.036 | ab | ab | ab | a | b |

*n* represents number of colonies included in measurement

* Insignificant differences in Dunn’s post-hoc pairwise comparisons after Bonferroni adjustment

**Table S3:** Timeline of the fall 2021 experiment in AB, detailing experimental day, actions involving experimental treatments, colony management and assessment actions, corresponding calendar dates, and sticky board collection interval.

| **Sticky Board** | **Experimental Day 2021** | **Treatment Actions** | **Colony Management Actions** | **Calendar Date 2021** | **Collection Interval (days)** |
| --- | --- | --- | --- | --- | --- |
| **1** | -4 |  | First assessment of food/brood, brood infestation, and worker bee population  Alcohol wash I | 30-Aug |  |
| **2** | 0 | Installation of 3c{3,6}, negative control, and Thymovar^®^ treatment applicators |  | 03-Sep | 4 |
| **3** | 1 |  |  | 04-Sep | 1 |
| **4** | 3 |  |  | 06-Sep | 2 |
| **5** | 5 |  |  | 08-Sep | 2 |
| **6** | 7 |  |  | 10-Sep | 2 |
| **7** | 14 |  |  | 17-Sep | 7 |
| **8** | 21 |  |  | 24-Sep | 7 |
| **9** | 28 | Removal of 3c{3,6} and Thymovar^®^ treatments  Clean-up phase and installation of Apivar^®^ begin | Second assessment of food/brood, brood infestation, and worker bee population  Alcohol wash II | 01-Oct | 7 |
| **10** | 29 |  |  | 02-Oct | 1 |
| **11** | 31 |  |  | 04-Oct | 2 |
| **12** | 33 |  |  | 06-Oct | 2 |
| **13** | 35 |  |  | 08-Oct | 2 |
| **14** | 42 |  |  | 15-Oct | 7 |
| **15** | 49 |  |  | 22-Oct | 7 |
| **16** | 56 |  |  | 29-Oct | 7 |
| **17** | 63 |  |  | 05-Nov | 7 |
|  | 70 | End of clean-up, removal of Apivar^®^ | Alcohol wash III  Final sticky board removed, no replacement | 12-Nov | 7 |

**Table S4:** Timeline of the fall 2021 experiment in BC, detailing experimental day, actions involving experimental treatments, colony management and assessment actions, corresponding calendar dates, and sticky board collection interval.

| **Sticky Board** | **Experimental Day 2021** | **Treatment Actions** | **Colony Management Actions** | **Calendar Date 2021** | **Collection Interval (days)** |
| --- | --- | --- | --- | --- | --- |
| **1** | -4 / -4 |  | First assessment of food/brood, brood infestation, and worker bee population  Alcohol wash I | 28-Aug |  |
| **2** | 0 | Installation of 3c{3,6}, negative control, and Thymovar^®^ treatment applicators |  | 01-Sep | 4 |
| **3** | 1 |  |  | 02-Sep | 1 |
| **4** | 2 |  |  | 03-Sep | 1 |
| **5** | 5 |  |  | 06-Sep | 3 |
| **6** | 7 |  |  | 08-Sep | 2 |
| **7** | 14 |  |  | 15-Sep | 7 |
| **8** | 21 |  |  | 22-Sep | 7 |
| **9** | 28 | Removal of 3c{3,6} and Thymovar^®^ treatments  Clean-up phase and installation of Apivar^®^ begin | Second assessment of food/brood, brood infestation  Alcohol wash II | 29-Sep | 7 |
| **10** | 39 |  |  | 30-Sep | 11 |
| **11** | 46 |  |  | 20-Oct | 7 |
| **12** | 56 |  |  | 27-Oct | 10 |
| **13** | 63 |  |  | 03-Nov | 7 |
|  | 70 | End of clean-up, removal of Apivar^®^ | Alcohol wash III  Final sticky board removed, no replacement | 10-Nov | 7 |

**Table S5:** Timeline of the fall 2022 experiment in AB, detailing experimental day, actions involving experimental treatments, colony management and assessment actions, corresponding calendar dates, and sticky board collection interval.

| **Sticky Board** | **Experimental Day 2022** | **Treatment Actions** | **Other Actions** | **Calendar Date 2022** | **Collection Interval (days)** |
| --- | --- | --- | --- | --- | --- |
| **1** | -5 |  | First assessment of food/brood, brood infestation, and worker bee population  Alcohol wash I | 08-Sep |  |
| **2** | 0 | Installation of 3c{3,6}, negative control, and Thymovar^®^ treatment applicators |  | 13-Sep | 5 |
| **3** | 1 |  |  | 14-Sep | 1 |
| **4** | 3 |  |  | 16-Sep | 2 |
| **5** | 5 |  |  | 18-Sep | 2 |
| **6** | 7 |  |  | 20-Sep | 2 |
| **7** | 14 |  |  | 27-Sep | 7 |
| **8** | 21 | Thymovar^®^ strips replaced |  | 04-Oct | 7 |
| **9** | 28 |  |  | 11-Oct | 7 |
| **10** | 35 |  | Second assessment of food/brood, brood infestation, and worker bee population | 18-Oct | 7 |
| **11** | 42 | Removal of 3c{3,6} and Thymovar^®^ treatments  Clean-up phase and installation of Apivar^®^ begin | Alcohol wash II | 25-Oct | 7 |
| **12** | 43 |  |  | 26-Oct | 1 |
| **13** | 45 |  |  | 28-Oct | 2 |
| **14** | 47 |  |  | 30-Oct | 2 |
| **15** | 49 |  |  | 01-Nov | 2 |
| **16** | 56 |  |  | 08-Nov | 7 |
| **17** | 63 |  |  | 15-Nov | 7 |
| **18** | 70 |  |  | 22-Nov | 7 |
| **19** | 77 |  |  | 29-Nov | 7 |
|  | 84 | End of clean-up phase, removal of Apivar^®^ | Alcohol wash III  Final sticky board removed, no replacement | 06-Dec | 7 |

**Table S6:** Timeline of the fall 2022 experiment in BC, detailing experimental day, actions involving experimental treatments, colony management and assessment actions, corresponding calendar dates, and sticky board collection interval.

| **Sticky Board** | **Experimental Day 2022** | **Treatment Actions** | **Other Actions** | **Calendar Date 2022** | **Collection Interval (days)** |
| --- | --- | --- | --- | --- | --- |
| **1** | -2 |  | First assessment of food/brood, brood infestation  Alcohol wash I | 12-Jul |  |
| **2** | 0 | Installation of 3c{3,6}, negative control, and Thymovar^®^ treatment applicators |  | 14-Jul | 2 |
| **3** | 1 |  |  | 15-Jul | 1 |
| **4** | 3 |  |  | 17-Jul | 2 |
| **5** | 5 |  |  | 19-Jul | 2 |
| **6** | 8 |  |  | 22-Jul | 3 |
| **7** | 15 |  |  | 29-Jul | 7 |
| **8** | 22 | Thymovar^®^ strips replaced |  | 5-Aug | 7 |
| **9** | 29 |  |  | 12-Aug | 7 |
| **10** | 36 |  |  | 19-Aug | 7 |
| **11** | 42 | Removal of 3c{3,6} and Thymovar^®^ treatments  Clean-up phase and installation of Apivar^®^ begin | Second assessment of food/brood, brood infestation  Alcohol wash II | 25-Aug | 6 |
| **12** | 43 |  |  | 26-Aug | 1 |
| **13** | 46 | Data missing for 3c{3,6} and negative control cardboard groups | | 29-Aug | 3 |
| **14** | 48 |  |  | 31-Aug | 2 |
| **15** | 50 |  |  | 02-Sep | 2 |
| **16** | 57 |  |  | 09-Sep | 7 |
| **17** | 64 |  |  | 16-Sep | 7 |
| **18** | 71 |  |  | 23-Sep | 7 |
| **19** | 78 | Data missing for all treatment groups | | 30-Sep | 7 |
|  | 84 | End of clean-up phase, removal of Apivar^®^ | Alcohol wash III  Final sticky board removed, no replacement | 06-Oct | 6 |
