# Supplementary material for: Field trials of the novel varroacide, 1-allyloxy-4-propoxybenzene, against Varroa destructor in Western Canada
Source: Sci Rep. 2025 Nov 17;15:40183. doi: 10.1038/s41598-025-23935-7 (PMC12623769; doi:10.1038/s41598-025-23935-7)
Supplement: Supplementary file 3 — Supplementary Material 3 [file 41598_2025_23935_MOESM3_ESM.docx]

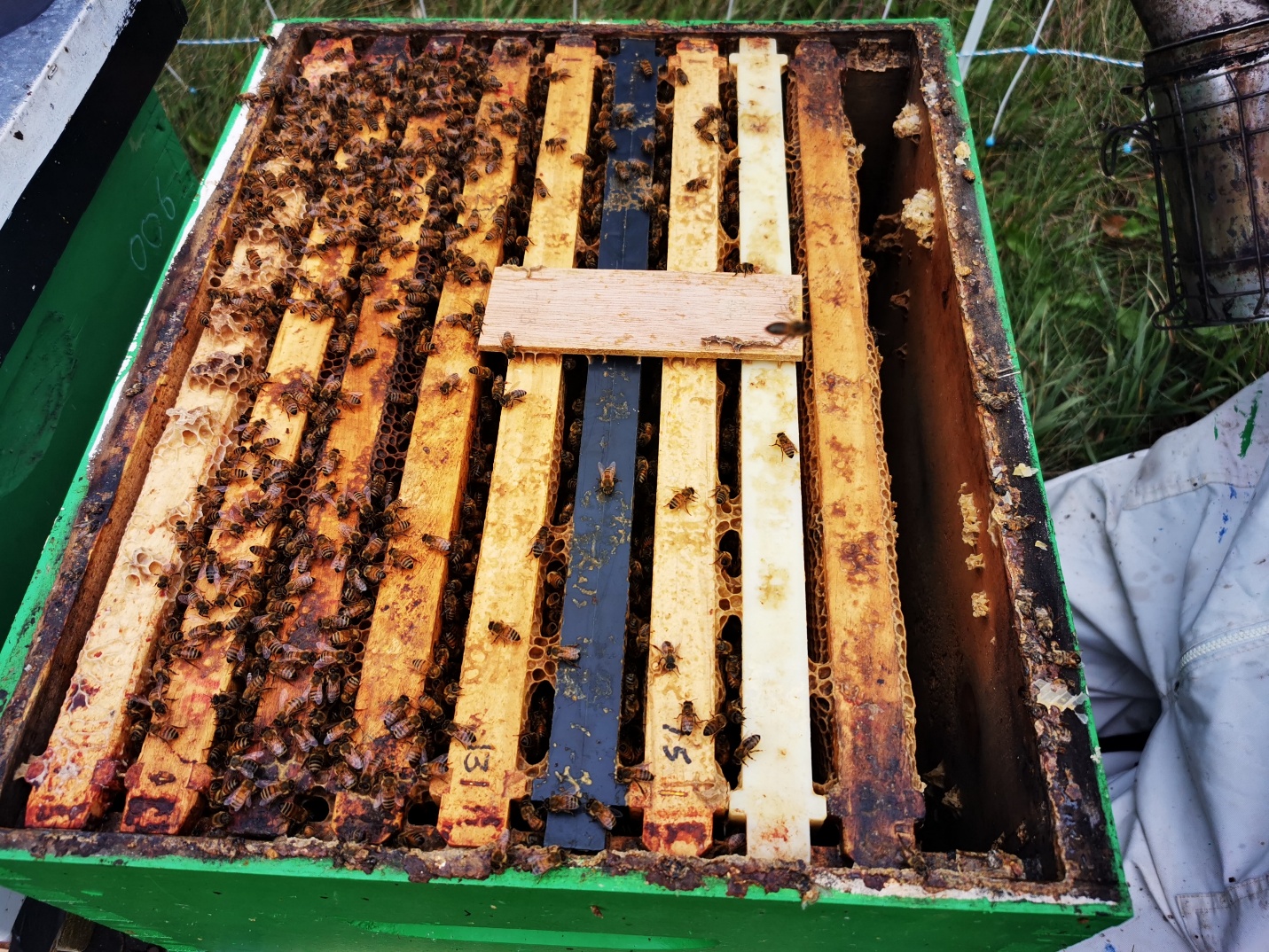


**Figure S1:** 3c{3,6} applicators used in the 2021 experiments, photographed within a colony undergoing varroacide treatment. The single 15.3 cm × 5.1 cm × 0.5 cm wooden strip was impregnated with 4 g compound 3c{3,6}, and placed centrally across the top bars of the broodnest as shown.


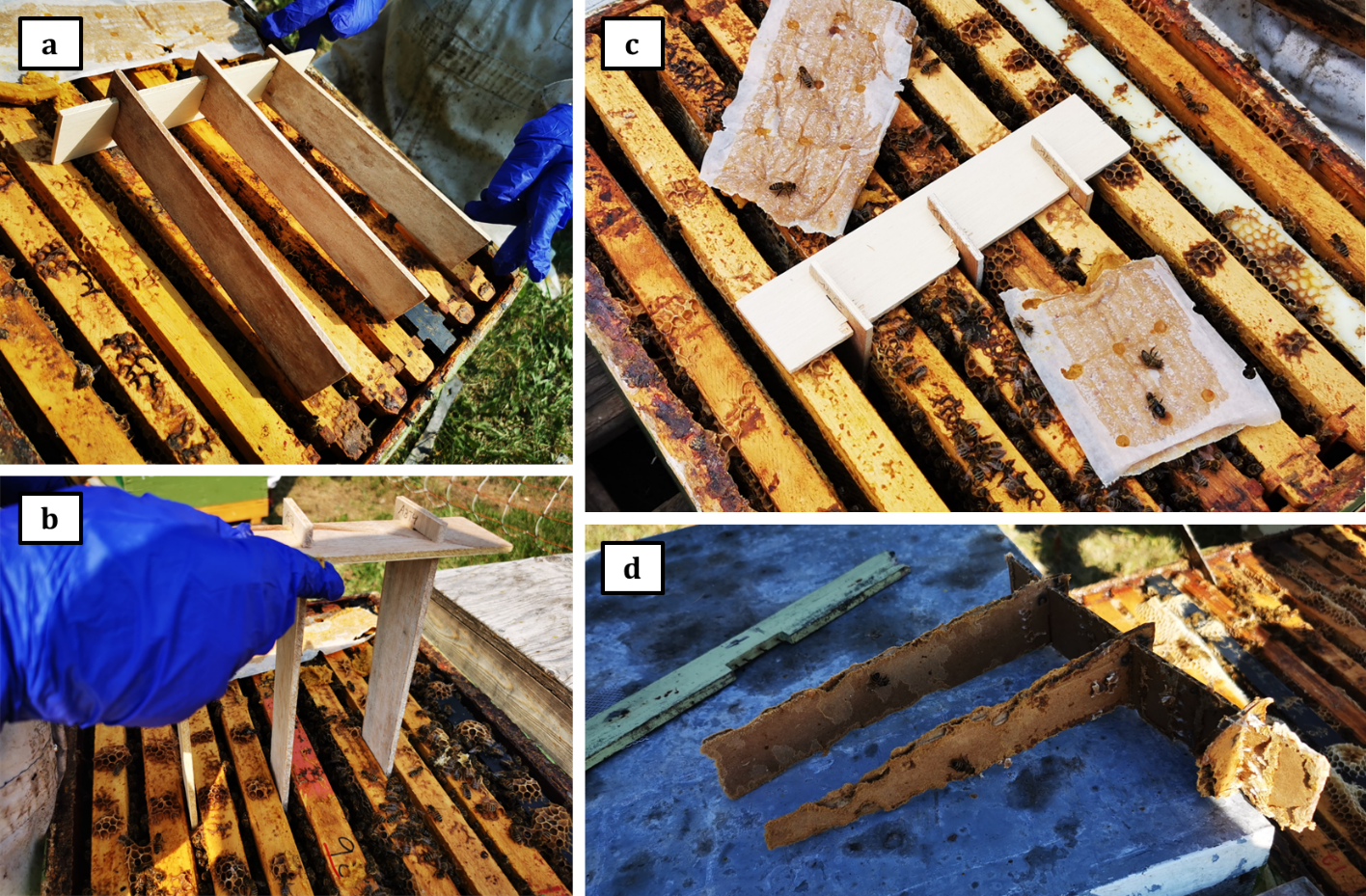


**Figure S2:** 3c{3,6} applicators used in the fall 2022 experiments. Three strips (24.0 cm × 5.0 cm × 0.5 cm wood, or 24.0 cm × 5.0 cm × 0.3 cm cardboard) were impregnated with a total of 8 g compound 3c{3,6}, and suspended between frames attached to a fourth untreated strip. [a] An uninserted wooden applicator, showing the general structure. [b] A wooden applicator being inserted into an experimental colony. [c] A fully-inserted wooden 3c{3,6} applicator, showing the general positioning of the three-strip applicators within colony broodnests. [d] A heavily-degraded cardboard 3c{3,6} applicator, having been removed following 42 days within the colony.
